# Comparative genomics of the *Pseudomonas corrugata* subgroup reveals high species diversity and allows the description of *Pseudomonas ogarae* sp. nov.

**DOI:** 10.1099/mgen.0.000593

**Published:** 2021-06-29

**Authors:** Daniel Garrido-Sanz, Miguel Redondo-Nieto, Marta Martin, Rafael Rivilla

**Affiliations:** ^1^​ Departamento de Biología, Facultad de Ciencias, Universidad Autónoma de Madrid, Darwin 2, 28049 Madrid, Spain; ^2^​ Department of Fundamental Microbiology, University of Lausanne, CH-1015 Lausanne, Switzerland

**Keywords:** *Pseudomonas corrugata*, *Pseudomonas ogarae*, phylogenomics, comparative genomics, PGPR

## Abstract

*Pseudomonas corrugata* constitute one of the phylogenomic subgroups within the *Pseudomonas fluorescens* species complex and include both plant growth-promoting rhizobacteria (PGPR) and plant pathogenic bacteria. Previous studies suggest that the species diversity of this group remains largely unexplored together with frequent misclassification of strains. Using more than 1800 sequenced *Pseudomonas* genomes we identified 121 genomes belonging to the *P. corrugata* subgroup. Intergenomic distances obtained using the genome-to-genome blast distance (GBDP) algorithm and the determination of digital DNA–DNA hybridization values were further used for phylogenomic and clustering analyses, which revealed 29 putative species clusters, of which only five correspond to currently named species within the subgroup. Comparative and functional genome-scale analyses also support the species status of these clusters. The search for PGPR and plant pathogenic determinants showed that approximately half of the genomes analysed could have a pathogenic behaviour based on the presence of a pathogenicity genetic island, while all analysed genomes possess PGPR traits. Finally, this information together with the characterization of phenotypic traits, allows the reclassification proposal of *Pseudomonas fluorescens* F113 as *Pseudomonas ogarae* sp. nov., nom rev., type strain F113^T^ (=DSM 112162^T^=CECT 30235^T^), which is substantiated by genomic, functional genomics and phenotypic differences with their closest type strains.

## Data Summary

The genomic and proteomic sequences used throughout this study are publicly accessible through the National Center for Biotechnology Information (NCBI), under the assembly accession numbers provided in File S1 (available in the online version of this article).

The authors confirm all supporting data, code and protocols have been provided within the article or through supplementary data files.

**Impact Statement**

The *Pseudomonas corrugata* subgroup within the *Pseudomonas fluorescens* species complex comprise both phytobeneficial and phytopathogenic species. Therefore, their use as inoculants in agriculture requires clear identification at the species level. Currently, there are only five recognized species, including *P. corrugata* and *P. mediterranea*, both described as phytopathogens. The analysis of a large number of sequenced genomes belonging to this group could provide a better understanding of how opposed lifestyles differ within these closely related species. Using genome-wide comparative genomic analyses, we have determined that the *P. corrugata* subgroup contains 29 species, most of them so far unacknowledged. PGPR traits are distributed across all the species groups, while a plant-pathogenic determinant is found in half of the genomes analysed. No plant-pathogenic determinants are found within the *P. brassicacearum*, *P. kilonensis, P.thivervalensis* and the newly described species *P. ogarae*, validating their use as agricultural inoculants. This new species type strain, F113^T^ (formerly *P. fluorescens* F113), is a model rhizobacterium for competitive root colonization and a well-known biocontrol agent. The results presented here also provide the basis for further analysis of the remaining uncharacterized species and expose an enormous bacterial diversity within the *P. corrugata* phylogenomic subgroup.

## Introduction

*Pseudomonas* is a highly diverse and metabolically versatile genus of *Gammaproteobacteria* [1] ubiquitously found in the environment. Members of this genus thrive from tropics to polar and desertic habitats [2–5] and have been found in fresh and sea waters [6, 7] and soils worldwide [8]. *Pseudomonas* can also colonize multiple niches, including humans, animals and plant tissues [9–12]. Among plant-associated *Pseudomonas*, both commensal and pathogenic lifestyles are reported across different species. For example, *P. syringae* is a known pathogen of a wide range of plant species [13]. Other plant pathogens include *P. corrugata* and *P. mediterranea* species, the causing agents of pith necrosis in tomato [14]. In contrast, multiple *Pseudomonas* species are recognized for their beneficial plant role, known as plant growth-promoting rhizobacteria (PGPR), and are mainly found within isolates and species from the *Pseudomonas fluorescens* complex of species [15, 16], including *P. brassicacearum*, *P. chlororaphis* and *P. protegens* [17–19].

Phylogenetic analyses have divided this diverse genus into two main lineages: *P. aeruginosa* and *P. fluorescens* [15, 20]. The *P. fluorescens* lineage is composed of three large groups: *P. fluorescens*, *P. syringae* and *P. putida*, commonly referred to as species complexes [15, 21, 22] due to the multiple species that are found within each of these groups. This complexity is further reflected in the number of different subgroups in which each of these larger groups can be subdivided. For instance, the *P. fluorescens* species complex is composed of nine distinct phylogenomic subgroups: *P. fluorescens*, *P. gesardii*, *P. fragi*, *P. mandelii*, *P. jessenii*, *P. koreensis*, *P. chlororaphis*, *P. protegens* and *P. corrugata* [15, 23]. The delineation of these different subgroups is supported by multilocus sequence analysis (MLSA), phylogenomics using average nucleotide identity (ANI) [24] and using the genome-to-genome blast distance phylogeny (GBDP) algorithm [25], which also digitally replicates the DNA–DNA hybridization ‘gold standard’ for species delineation and therefore provides a robust and accurate delineation of species and phylogenomic groups [15, 26]. In addition, comparative genomic analysis of the *P. fluorescens* species complex has shown that certain traits involved in the PGPR and biocontrol abilities of strains from these subgroups follow a phylogenomic distribution [15].

The *P. corrugata* subgroup within the *P. fluorescens* species complex is composed of five named species: *P. corrugata*, *P. mediterranea*, *P. thivervalensis*, *P. kilonensis* and *P. brassicacearum* [15, 23], although the number of species of this subgroup is probably higher, as suggested in multiple studies [15, 26–28], which also emphasize frequent misclassification of strains. In addition, the *P. corrugata* subgroup is particularly baffling, since it includes recognized PGPR members together with plant-pathogenic species and strains. Among the PGPR traits, the production of the antibiotic 2,4-diacetylphloroglucinol (DAPG) and 1-aminocyclopropane-1-carboxylic acid (ACC) deaminase are probably the most studied characters within PGPR pseudomonads. The biosynthesis of the broad-spectrum antibiotic DAPG [29] has also shown to elicit the plant-induced systemic resistance [30] and stimulate lateral root growth by interacting with the signalling pathway of the phytohormone auxin [31]. On the other hand, ACC deaminase contributes to phytostimulation by promoting root growth [32] and also alleviate abiotic stresses [33, 34]. Conversely, the *P. corrugata* subgroup also includes the causal agents of pith necrosis in tomato, *P. corrugata* [35], which gives the name to this subgroup by oldest species description, and *P. mediterranea* [36], together with other plant pathogens, including *Pseudomonas* sp. N2C3, which kills or stunt plants from the families *Brassicaceae* and *Papaveroideae* [37]. Within the plant-associated pathogenic pseudomonads of this subgroup, a genetic island containing non-ribosomal peptide synthetase genes and genes similar to the acyl-homoserine lactone quorum-sensing system in *Proteobacteria* [named lipopeptide/quorum sensing (LPQ) island] is found and reportedly responsible for their plant-pathogenic performance [37, 38]. Surprisingly, *P. corrugata* is used for the biocontrol of a number of plant-pathogenic bacteria and fungi [39], and they also harbour multiple PGPR traits, including hydrogen cyanide and auxin biosynthesis [27]. This ambivalent behaviour could be explained by other parameters, such as the genotype and physiological state of the plant or the ability of bacteria to manipulate the plant immune system [27].

In this work, we analysed more than a hundred genomes belonging to the *P. corrugata* subgroup within the *P. fluorescens* species complex in order to determine their current species status. Using phylogenomics and comparative and functional genomics we determined a high number of species groups that were previously unacknowledged. Using a polyphasic approach we propose the reclassification of *P. fluorescens* F113 into the novel species *P. ogarae* sp. nov., type strain F113, which is substantiated by phylogenomics, functional genomics and phenotypic analyses.

## Methods

### Identification of genomes belonging to the *P. corrugata* subgroup

From the 10 785 *Pseudomonas* genomes listed in the NCBI RefSeq database in February 2020, those of species and lineages outside *P. fluorescens* phylogenetic groups (as previously described in [15]) were removed. These include genomes assigned to *P. aeruginosa*, *P. putida*, *P. syringae* complex of species and *P. stutzeri*. The remaining 1811 *Pseudomonas* genomes were downloaded from the NCBI RefSeq database in February 2020.

Genomes belonging to the *P. corrugata* phylogenomic subgroup were determined based on the GBDP distance threshold of 0.132915 previously established for the *P. fluorescens* complex of species [15] using the genome of *P. ogarae* F113^T^ (this study, formerly *P. fluorescens* F113) as a reference, which has been previously classified into the *P. corrugata* subgroup in several previous studies [15, 26, 40, 41]. Overall, 121 genomes were shown to belong to the *P. corrugata* subgroup (File S1) and therefore were selected for further analyses.

### Genome sequencing, assembly and annotation of *P. ogarae* RDP1

*P. ogarae* RDP1 was isolated from the rhizosphere of pepper (*Capsicum annuum*) as previously described [26]. Total DNA was extracted using the Realpure Genomic DNA extraction Kit (Durviz, Spain) and sequenced by paired-end Illumina MiSeq 2×150 at Parque Científico de Madrid (Spain). Reads were quality-filtered using Trimmomatic software v0.38 [42]. The genome of *P. ogarae* RDP1 was assembled using SPAdes v3.14.1 software [43], read error correction and careful parameters. Prokka v1.14.6 software [44] was used to annotate the genome, using the genetic code number 11 and default parameters. The genome sequence has been deposited in the NCBI and it is publicly available under the BioProject accession number PRJNA697838.

### Phylogenomic analysis and clustering

Intergenomic distances among the 121 identified *P. corrugata* subgroup genomes and representative type strain sequenced genomes of the remaining *P. fluorescens* subgroups (*P. mandelii* subgroup: *P. mandelii* LMG 21607^T^ and *P. lini* CCUG 51522^T^; *P. jessenii* subgroup: *P. jessenii* DSM 17150^T^ and *P. vancuoverensis* CCUG 49675^T^; *P. koreensis* subgroup: *P. koreensis* CCUG 51519^T^ and *P. baetica* LMG 25716^T^; *P. chlororaphis* subgroup: *P. chlororaphis* subsp. *chlororaphis* DSM 50083^T^; *P. protegens* subgroup: *P. protegens* CHA0^T^; *P. fluorescens* subgroup: *P. veronii* DSM 11331^T^, *P. azotoformans* DSM 18862^T^, *P. fluorescens* ATCC 13525^T^ and *P. synxantha* DSM 18928^T^; *P. gesardii* subgroup: *P. gesardii* DSM 17152^T^ and *P. mucidolens* NBRC 103159^T^; *P. fragi* subgroup: *P. fragi* NBRC 2458^T^) and type strains genomes of other *Pseudomonas* main groups (*P. aeruginosa* ATCC 10145^T^, *P. resinovorans* DSM 21078^T^, *P. putida* NBRC 14164^T^ and *P. syringae* KCTC 12500^T^) were calculated using the GBDP algorithm [25], via the genome-to-genome distance calculator (GGDC: http://ggdc.dsmz.de/) web service. The obtained sets of intergenomic distances (File S2) were transformed into a matrix and imported to mega X software [45] to build the neighbour-joining (NJ) phylogenomic tree. *Escherichia coli* ATCC 11775^T^ was used as the outgroup. GBDP was also used to calculate digital DNA–DNA hybridization (dDDH) values among all genome pairwise comparisons. In addition, the Type (Strain) Genome Server (TYGS: https://tygs.dsmz.de/) web service was used with the identified novel unnamed species-level groups for a whole genome-based taxonomic analysis [46], using default parameters.

Clustering of intergenomic distances at species level (dDDH≥70 %) and into phylogenomic groups was examined using the OPTSIL clustering software v1.5 [47] as previously described [48]. Briefly, distance threshold (*T*) values from 0 to 0.2, using a step size of 0.0005 were evaluated using single-, average- and complete-linkage clustering (*F*=0, 0.5 and 1, respectively). The best *T* and *F* for both, species-level and phylogenomic group clusters were chosen based on the highest modified Rand Index (MRI) score.

### Orthologous groups identification and genome fractions

Proteomes of the 121 genomes belonging to the *P. corrugata* subgroup were analysed with OrthoFinder software v2.3.3 [49], using diamond v0.9.131 [50] searches and the MCL graph-clustering algorithm [51]. Resulting orthologous groups (OGs) were analysed with a previously designed R script [48] to obtain the core, group-specific and pan genome fractions over 1000 randomly sampled genomes. The mean, Q1 and Q3 statistics were represented using the ggplot2 R package [52].

### Phylogeny of single-copy genes

Orthologous sequences of 1891 single-copy genes present in the 121 *P*. *corrugata* subgroup genomes were aligned with Clustal Omega [53] and further concatenated. The resulting concatenated alignment was examined with gblocks v0.91 [54] to remove poorly aligned columns and highly divergent regions that might not be homologous or may have been saturated by multiple substitutions [55], using a minimum block length of two amino acids. The resulting matrix was imported into the RaxML-NG v0.9.0 [56] to build a maximum-likelihood (ML) phylogenetic tree, using the LG model of amino acid evolution [57] combined with gamma-distributed substitution rates and empirical frequencies of amino acids. Fast bootstrap and subsequent search for the best-scoring tree [58] and the autoMRE criterium [59] were applied.

### Functional annotation of orthologs and distribution

The functional annotation of orthologous groups was achieved using eggNOG-Mapper v2.0 [60] based on eggNOG v.5.0 orthology data [61]. Sequence searches were performed using diamond. KEGG BRITE hierarchical classification was retrieved using the KEGGREST R package [62] and represented using ggplot2. In addition, the UpSet R package [63] was used to represent specific intersections of the orthologous groups’ matrix and aggregate number of grouped elements.

A principal component analysis (PCA) was performed with the prcomp function of the stats R package, using the relative abundance of the last-rank BRITE categories assigned to OGs in each genome (considering singletons and paralogues) as input variable. Forty-four genomes consisting of more than 75 contigs (File S1) were discarded to avoid misrepresentation of categories. Separation of PCA observation groups was measured and quantified as previously described [64]. Briefly, Mahalanobis distances [65] between groups, using the first two principal components’ centroids of the PCA were used to calculate Hotelling’s two-sample *T^2^* statistic, which was then used to obtain the *F*-value. Finally, *F*-critical values were calculated using an *α*=0.05 and compared against the obtained *F*-value for significance. All the mathematical formulas applied are specified in [64].

### Phenotypic characterization of *P. ogarae*, *P. kilonensis, P. brassicacearum* and *P. thivervalensis*

Type strains of *P. brassicacearum* DSM 13227^T^, *P. thivervalensis* DSM 13194^T^ [66] and *P. kilonensis* DSM 13647^T^ [67] were acquired from the German Collection of Microorganisms and Cell Cultures (DSMZ). *P. ogarae* F113^T^ (this study, formerly *P. fluorescens*) was first isolated by the group of O’Gara in 1992 [68] and received in our laboratory in 1997, where its whole genome was sequenced [69]. The four strains together with *P. ogarae* RDP1 (this study), *Pseudomonas* sp. WCS365, *P. brassicacearum* NFM421 and *P. fluorescens* Q8r1-96 (the three belonging to *P. brassicacearum* species) were characterized at the phenotypic level by the determination of enzymatic activities and oxidation of carbon substrates by using API 20 NE and API 50 CHB/E (bioMérieux). API tests were done following the manufacturer specifications and read after 48 h of incubation at 28 °C. Assimilation of carbon substrates was determined using Biolog GEN III MicroPlate (Biolog) with some modifications. Fresh (overnight growth) cells were resuspended in 10 ml of minimal medium [70] until an optical density at 600 nm (OD_600_) of 0.05 was reached. In total, 100 µl of each cell resuspension was transferred into the plate’s wells and incubated at 28 °C in a rotary shaker (135 r.p.m.) for 16 h. Assimilation of carbon substrates was evaluated as the ability of the cells to grow by measuring the OD_600_ and its comparison to the negative control for the assimilation tests and the positive control for Biolog’s sensitivity assays. Positive results were considered when the DO_600_ obtained was at least twice that of the negative control (for assimilation) and at least 75 % of the positive control (for sensitivity).

A non-metric multidimensional scaling (NMDS) ordination analysis was used to plot the dissimilarity of the strains tested based on API and Biolog results. A positive result was given a value of 1, a negative result a value of 0 and a weak growth a value of 0.5. Only characters that show differences among the strains were selected. NMDS ordination analysis based on Jaccard distances were calculated using the metaMDS function of the vegan R package [71], using a number of dimensions, *k*=2.

*P. ogarae* F113^T^ (this study, formerly *P. fluorescens*) was deposited in the DSMZ culture collection and the Spanish Type Culture Collection (CECT) under the collection numbers DSM 112162^T^ and CECT 30235^T^, respectively.

## Results and discussion

### Phylogenomic analysis and clustering of *P. corrugata* subgroup genomes

**Fig. 1.**
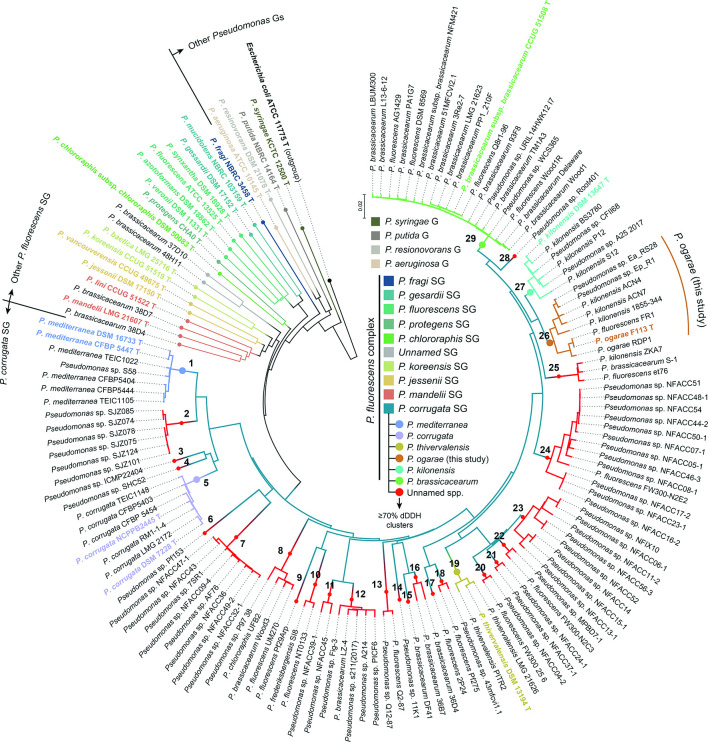
GBDP-based phylogeny of 121 genomes belonging to *P. corrugata* and other major subgroups within the *P. fluorescens* complex of species and other major *Pseudomonas* groups. Colours according to the phylogenomic group (G), subgroup (SG) or species identified by clustering analyses of GBDP intergenomic distances (Fig. 2, File S3). Numbers in nodes leading to species-clusters are used throughout the study. Bold T, and coloured taxa name indicate type strain.

**Fig. 2.**
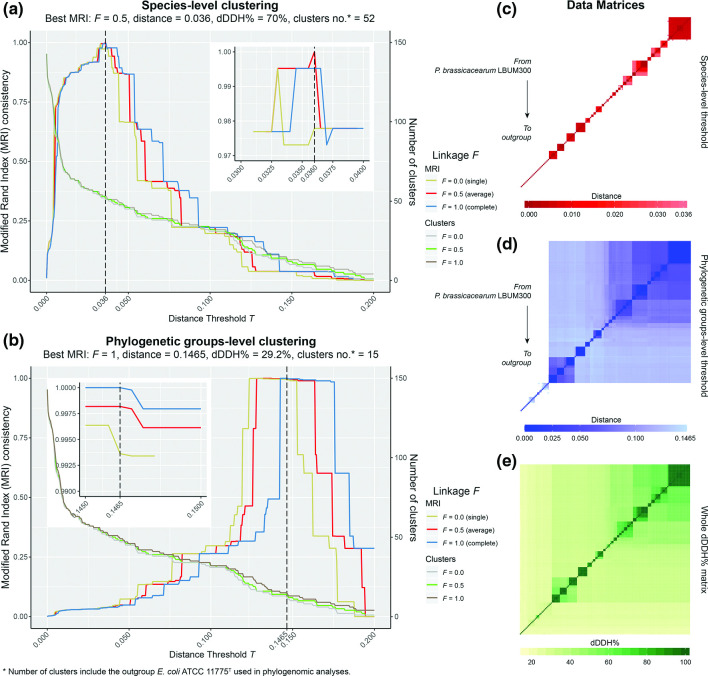
Clustering analysis of 145 genomes belonging to the *P. corrugata* subgroup and the other main subgroups and groups within the *Pseudomonas* genus (Fig. 1) using a range of distance thresholds *T*. Total cluster consistency (i.e. MRI=1) was achieved using an average linkage (i.e. *F*=0.5) at species-level (a) or complete linkage (i.e. *F*=1) at phylogenomic group-level (b) compared to the reference partition. Distance matrices (c, d) and digital DNA–DNA hybridization (dDDH) matrix (e), showing these clusters.

The 121 genomes identified as putatively belonging to the *P. corrugata* subgroup, together with representative genomes of the remaining *P. fluorescens* subgroups and other major *Pseudomonas* groups were used to calculate intergenomic distances and digital DNA–DNA hybridization values using the GBDP algorithm [25]. These distances were further used to build a phylogenomic tree and for clustering analysis. The phylogenomic tree obtained (Fig. 1) agrees with accepted phylogenies of *Pseudomonas* and the *P. fluorescens* complex of species [15, 23, 72]. The clustering analysis (Fig. 2, File S3) shows that, at the phylogenomic-group level, a distance threshold of 0.1465 and using a complete-linkage clustering, results in total consistency (i.e. MRI=1) with the reference partition, unequivocally assigning 121 genomes as belonging to the *P. corrugata* subgroup and discriminating the remaining *Pseudomonas* subgroups. The distance threshold obtained is slightly higher than the one previously established for eight *P. fluorescens* subgroups. In a previous study, using only 93 genomes belonging to the *P. fluorescens* complex of species, the authors identified an intergenomic distance threshold of 0.132915 [15], while here we report a distance of 0.1465 as the threshold for genomes belonging to the *P. corrugata* subgroup. These differences could be due to the larger dataset used here, which allows a more precise threshold determination, and to the fact that one subgroup and a single representative genome of each of the remaining *P. fluorescens* subgroups have been considered. Nonetheless, this value threshold assigned 121 *Pseudomonas* genomes over more than *c.a*. 1800 genomes, as belonging to the *P. corrugata* subgroup (File S1).

At the species level, the clustering of intergenomic distances revealed a far greater diversity than previously thought. At present, there are only five validly named species within the *P. corrugata* subgroup: *P. brassicacearum*, *P. kilonensis*, *P. thivervalensis*, *P. corrugata* and *P. mediterranea* [15, 23, 72]. However, based on the clustering of the intergenomic distances at a threshold *T*=0.036, which equals 70 % of digital DNA–DNA hybridization (dDDH, i.e. the ‘gold standard’ for species delineation), there are 29 species clusters within the *P. corrugata* subgroup (Fig. 1), yielding total consistency (i.e. MRI=1) with the reference partition when using an average-linkage clustering (i.e. *F*=0.5, Fig. 2a). Among them, the largest species-cluster is *P. brassicacearum* (group 29, Fig. 1), composed of 19 genomes, including the type strain CCUG 51508^T^, while eight species-level clusters contain only one representative genome.

Surprisingly, there are multiple genomes misclassified at the species level. These include *P. brassicacearum* strains S-1, 36D4, 36B7, DF41, Lz-4 and Wood3, and four others that do not even belong to the *P. corrugata* subgroup: 28D4 and 38D7 within the *P. mandelii* subgroup, and 48H11 and 37D10 strains that according to our analysis consist in a novel phylogenomic group within the *P. fluorescens* complex of species (Fig. 1), *P. kilonensis* strains ACN4, ACN7, 1855–344 and ZKA7 that also do not cluster together within the type strain *P. kilonensis* DSM 13647^T^, and numerous strains wrongly assigned to *P. fluorescens*: AG1429, DSM 8569, Q8r1-96, Wood1R, FR1, F113 (*P. ogarae* this study, formerly *P. fluorescens*), et76, FW300-N2E2, FW300-N2C3, FW300_25_6, Pf275, 2P24, Q2-87, NT0133, Pf29Arp and UM270. There are also two strains wrongly assigned to *P. frederiksbergensis* SI8 and *P. chlororaphis* UFB2. Nonetheless, the majority of genomes belonging to the *P. corrugata* subgroup are still unclassified at the species level.

In addition, the Type (Strain) Genome Server (TYGS) [46], was used for a whole genome-based taxonomic analysis to further evaluate the 29 species-level clusters identified. The results are shown in the File S4 and validate species status for all the species-level clusters identified in this study. Only strain genomes clustered within the five named species (*P. brassicacearum*, *P. kilonensis*, *P. thivervalensis*, *P. corrugata* and *P. mediaterranea*, Fig. 1) belong to these species, while the remaining clusters at the species level represent novel species.

One of these novel species cluster includes, among others, a widely used PGPR model of plant-root colonization: *Pseudomonas ogarae* F113^T^ (this study, formerly *P. fluorescens* F113, F113 hereafter), isolated in 1992 from the rhizosphere of sugar beet [68]. Although F113 was one of the first genomes belonging to the *P. fluorescens* complex of species to be completely sequenced [69], it is long known that F113 indeed does not belong to the *P. fluorescens* species [15, 26, 40]. A previous study tried to reclassify this strain as belonging to *P. kilonensis* [40]. Nonetheless, our analysis evidence that neither *P. fluorescens* nor *P. kilonensis* is the proper species name of F113, which constitutes a novel species together with strain RDP1 (this study), unclassified isolates Ea_RS28 and Ep_R1, misclassified *P. kilonensis* strains ACN4, ACN7 and 1855–344, and misclassified *P. fluorescens* strain FR1 (Fig. 1). Indeed, the comparison of F113 against *P. kilonensis* DSM 13647^T^ achieved a dDDH of 64.5 % (confidence interval of 61.6–67.3 %, File S2), clearly demonstrating that F113 does not belong to *P. kilonensis* species. This was also substantiated by the TYGS analysis, which categorically shows that the eight mentioned strains belong to the same novel species, with no type strain of any other described species close enough (File S4) among more than 12000 type strains currently included in the server. Among the 228 *Pseudomonas* species validly published to date (August 2020), the TYGS server included 201. The remaining 27 *Pseudomonas* type strains not included in the TYGS server do not belong to the *P. corrugata* subgroup according to 16S rRNA, MLSA, whole-genome analysis or a combination of these analyses (File S5).

### Single-copy proteins phylogeny and genome fractions

**Fig. 3.**
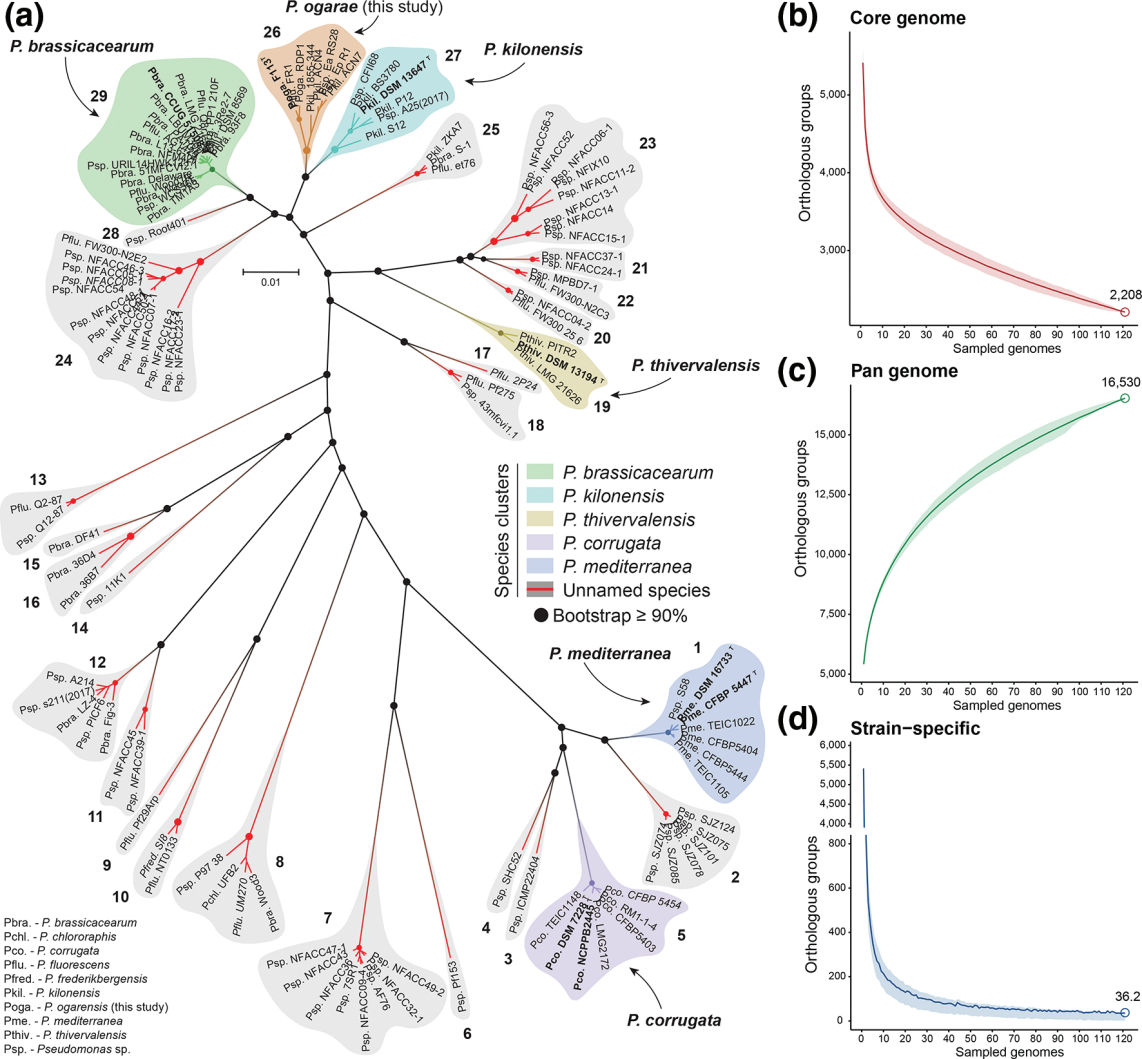
Analysis based on the identification of orthologous groups. (a) Unrooted maximum-likelihood tree of 1891 single-copy amino acid sequences present in the 121 genomes belonging to the *P. corrugata* subgroup. Bold and ^T^ indicate type strain. Species groups are shadowed in colour according to the legend and numbers according to Fig. 1. Dots indicate bootstrap support ≥90 % (not shown in nodes within a species cluster). Core genome (b), pan genome (c), and strain-specific genome (d) fractions over the sampled genomes. Mean values (line) and Q1 and Q3 quantiles (shadow) over 1000 replicates of randomly sampled genomes are represented. Empty circles and the number above indicate the mean genome fraction value achieved at 121 genomes sampled.

The determination of orthologous groups of amino acid sequences among the 121 *P*. *corrugata* subgroup genomes identified 1891 single-copy proteins present in all the genomes. These single-copy proteins were used to build a maximum-likelihood phylogenetic tree based on their alignment and concatenation. The results (Fig. 3a) show a clustering pattern identical to that previously observed based on whole-genome analysis at species-level, with bootstrap support ≥90 % in the nodes leading to these clusters. This result further validates the presence of 29 species within the *P. corrugata* subgroup. Nonetheless, the relative position of certain clusters differs compared to the phylogenomic tree based on intergenomic distances (Fig. 1). For instance, group 24 (composed of 13 unclassified isolates) is closer to *P. brassicacearum* in the core-proteins' tree (Fig. 3a) than in the whole-genome phylogeny (Fig. 1), which could imply a different evolutive pressure of the core genome versus the whole genome or a bias in codon usage.

The core genome, pan genome and strain-specific genome were also determined based on the number of orthologous groups present in all genomes (core genome), the number of different orthologous groups (pan genome), and the novel orthologous groups (strain-specific genome) that appear with the addition of randomly sampled genomes (Fig. 3b–d). Regarding the core genome, with 121 genomes it is composed of 2 208 orthologous groups. As expected, the core genome of the *P. corrugata* subgroup is smaller than the previously estimated (3438) based only on 12 genomes [15]. It is also considerably smaller than the core genome of only five *P. corrugata* and four *P. mediterranea* genomes, consisting of 4 469 orthologous groups [14], probably due to a misrepresentation of the whole genetic diversity of this subgroup. On the other hand, the core-genome curve (Fig. 3b) shows a still decreasing slope, which indicates that it has not yet achieved a full representation of the subgroup core diversity. Similarly, the ‘open’ pan genome (Fig. 3c) of 16 530 orthologous groups suggests a higher genetic diversity yet to be discovered. Conversely, the strain-specific curve (Fig. 3d) shows that around 20 genomes are enough to cover most of the shared diversity and each genome adds an average of 36 novel orthologous groups. This could indicate a high degree of genetic homogeneity among closely related clusters.

### Overall functional diversity of *P. corrugata* species groups

**Fig. 4.**
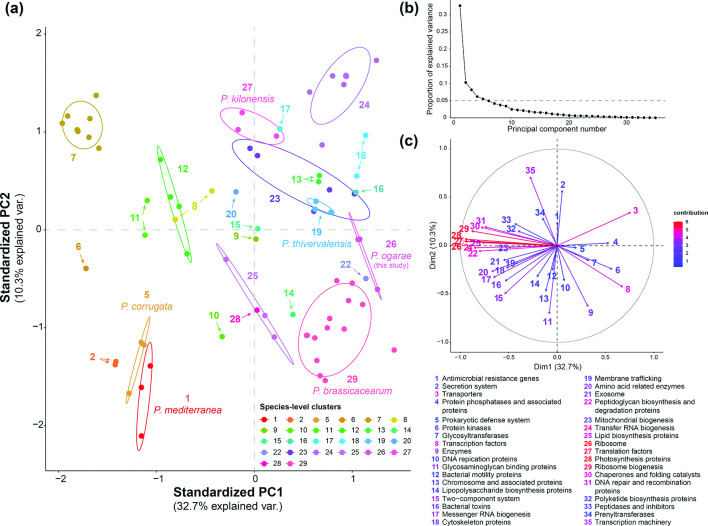
Principal component analysis (PCA) of functional BRITE categories among *P. corrugata* subgroup genomes. (a) Biplot of the first two principal components (PC) explaining the 43 % of the observed variance. The different groups are coloured according to the legend. Ellipses represent the core area added by the default confidence interval of 68 % to facilitate the separation between observation groups. (b) Proportion of explained variance by each PC. Dashed horizontal line indicates 5 % of explained variance. (c) Principal component variables, zero-centred and scaled to unit variance.

To further evaluate if the differences observed at the species groups based on intergenomic distances and the core-proteins also extend to the functional content of the groups, we performed a principal component analysis (PCA) using the BRITE categories assigned to the orthologous groups, previously calculated, as variables. To avoid misrepresentation of categories due to incomplete genomes, those with more than 75 contigs were removed from the analysis. The results show a clear distinction of genomes belonging to the same species-level cluster (Fig. 4a). The first two principal components explain the 43 % of observed variance (32.7 % PC1 and 10.3 % PC2, respectively) and clearly distinguish *P. corrugata* and *P. mediterranea* pathogenic species [35, 36] from known PGPR, including *P. brassicacearum* (group 29 [17]) and *P. ogarae* (group 26 [32]). Interestingly, certain strains that have been shown to be plant pathogens, including N2C3 (group 22 [37]), are functionally more closely related to other PGPR groups than to *P. corrugata* and *P. mediterranea* (Fig. 4). This finding agrees with the observation of plant pathogens within the *P. corrugata* subgroup also harbour multiple PGPR traits, including hydrogen cyanide and auxin biosynthesis [27], and could indicate that traits relevant for pathogenesis are not the only driving feature of certain groups. In fact, it is known that the pathogenicity of *P. corrugata*, *P. mediterranea* and other strains from the *P. corrugata* subgroup is associated with a genetic island [37] and, more interestingly, this pathogenic ability only occurs towards certain plant species, while in others these bacteria act as PGPR/biocontrol agents [27, 39]. Another interesting group (group 7), composed of eight unclassified isolates, clearly diverges in its functional content from the rest of the observation groups (Fig. 4), which suggests the presence of specialized and distinctive traits.

Statistical analysis of the first two PCs’ centroids demonstrates that most PCA observation groups are significantly different (File S6). Specifically, *P. ogarae* (group 26), is significantly different at the functional level compared to their closest species: *P. thivervalensis*, *P. kilonensis* and *P. brasicacearum* (groups 19, 27 and 29, respectively), also significantly different among them, which substantiate the differences found at the genomic level and further support their status as different species. Interestingly, the closely related species *P. mediterranea* and *P. corrugata* (groups 1 and 5, respectively) were not found to be statistically different at the functional level among the first two PCs (File S6). This could be related to subtle differences that are not represented by the functional categories assessed. It is also important to notice that certain genomes were discarded from the functional analysis given that they contained more than 75 contigs. Therefore, the groups represented in the PCA only account for a fraction of genomes that might not be enough to reflect their total diversity. In addition, only the first two PCs were used for the statistical analysis, which accounts for 43 % of the explained variance.

Regarding the specific BRITE functional categories that explain the variance of the observation groups, in the case of PGPR species, transporters and transcription factors are the variables that contribute the most to the observed variance, followed by protein kinases and secretion systems (Fig. 4c). In contrast, the variance of *P. corrugata* and *P. mediterranea* species are driven by two-component systems, bacterial toxins, and amino acid-related enzymes variables, among others. In the specific case of *P. ogarae* (group 26), prokaryotic defence systems, protein kinases and glycosyltransferases positively correlate with this group, while antimicrobial resistance genes and secretion systems contribute to *P. kilonensis* species group.

In addition, the search for genes involved in PGPR traits and the LPQ genetic island responsible for the pathogenicity of certain species and strains from the *P. corrugata* subgroup [37] shows an interesting distribution pattern. As shown in File S7 (Fig. S1), 49 genomes harbour the LPQ island. These include all genomes from *P. mediterranea, P. corrugata* and species clusters numbers 2, 3, 4, 6, 7, 14, 15, 16, 20, 21, 22, 25 and 28, and it is partly present in genomes from the species-cluster 23 (clusters numbers according to Fig. 1). Conversely, this LPQ island is absent from *P. brassicacearum*, *P. kilonensis*, *P. ogarae* and *P. thivervalensis* species clusters (File S7). However, certain PGPR traits, such as the biosynthesis of the antibiotic DAPG, ACC deaminase, biosynthesis of hydrogen cyanide and genes for denitrification, among others, are found in genomes harbouring the LPQ island and those in which this island is absent. This observation was not unexpected, as it has been previously reported [27].

### Functional differences of *P. brassicacearum*, *P. kilonensis* and *P. ogarae* type strains

**Fig. 5.**
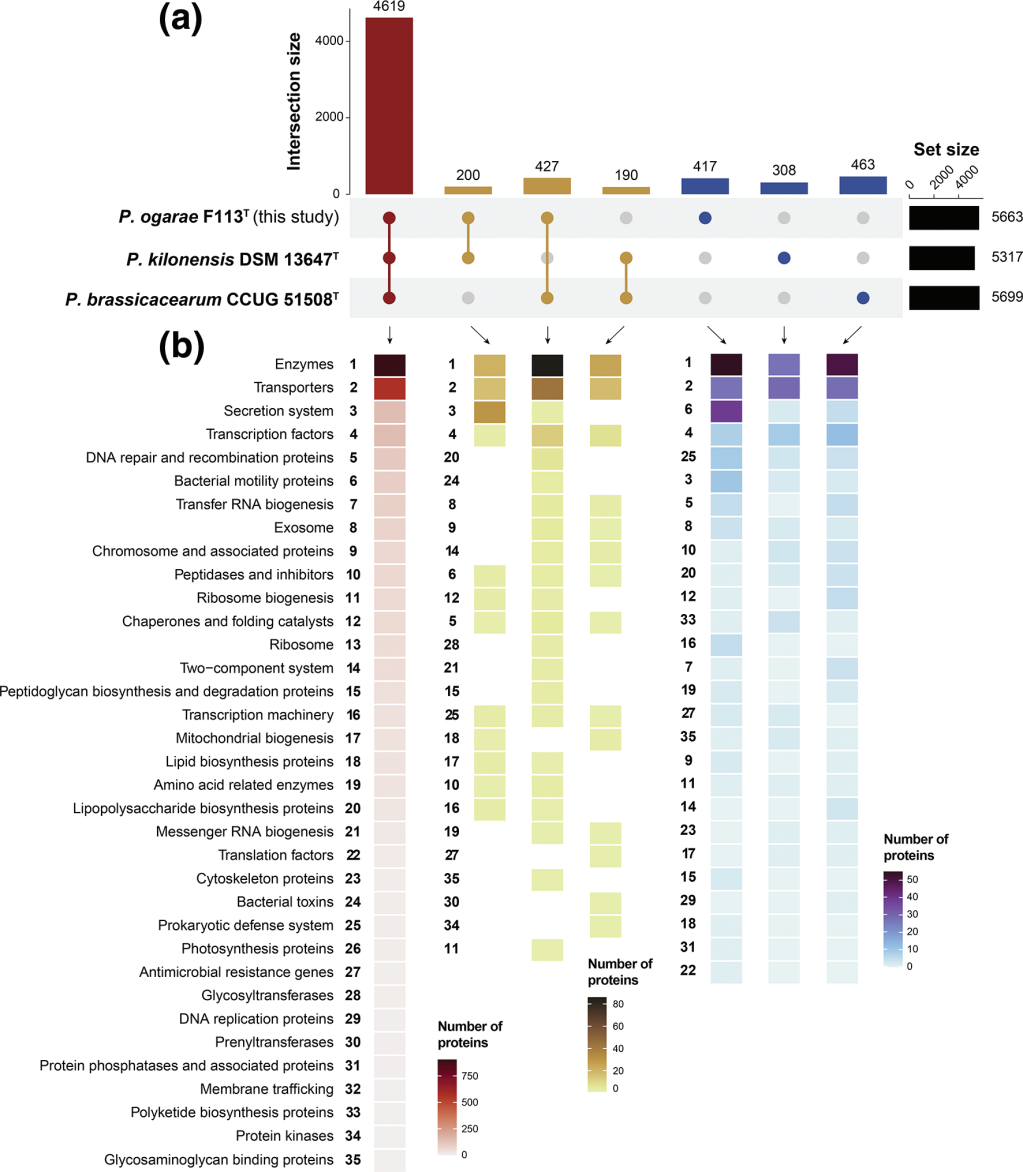
Comparative analysis of closely related *P. brassicacearum*, *P. kilonensis* and *P. ogarae* type strains genomes based on orthologous sequences. (a) Upset plot showing the intersections of shared orthologous proteins among the three genomes (red), the shared proteins between each two genomes (black) and specific proteins to each genome (blue). (b) Abundance of BRITE categories among the different intersections.

Type strains are defined as the nomenclatural type of a new taxon used for description, as defined by the Bacteriological Code [73], that ‘exhibit all of the relevant phenotypic and genotypic properties cited in the original published taxonomic circumscriptions’ [74]. Therefore, we used the type strain genomes of *P. brassicacearum* CCUG 51508^T^ [66] and *P. kilonensis* DSM 13647^T^ [67], together with *P. ogarae* F113^T^ [68], the most studied strain of this novel species, to identify differences in their functional content based on the identification of orthologous sequences and BRITE classification of proteins. The results are summarized in Fig. 5 and discussed below.

The three genomes analysed, type strains of *P. kilonensis*, *P. brassicacearum* and *P. ogarae* (this study), share 4629 proteins, representing between 81–86.9 % of their respective total protein content. Most of these shared proteins are classified in enzymes and transporters BRITE categories. However, there are more proteins common to *P. ogarae* and *P. brassicacearum* (427) than any other combination (200 between *P. ogarae* and *P. kilonensis* and 190 between *P. kilonensis* and *P. brassicacearum*). This higher functional similarity between *P. ogarae* and *P. brassicacearum* was previously observed in the PCA (Fig. 4a) and contrasts with the phylogenomic analysis, in which *P. kilonensis* and *P. ogarae* species groups are more closely related to one another than to *P. brassicacearum* (Fig. 1). The 427 proteins shared between *P. ogarae* and *P. brassicacearum* are classified into 22 distinct categories, and are mostly enzymes, transporters and transcription factors and, to a less extent, lipopolysaccharide biosynthetic proteins, bacterial toxins, glycosyl-transferases, peptidoglycan biosynthesis proteins and glycosaminoglycans binding proteins, which are not shared by the other genome combinations (Fig. 4b). This suggests that both *P. ogarae* and *P. brassicacearum* might form similar membrane and extracellular matrix components, which are crucial to form biofilms and to colonize the rhizosphere [75, 76]. Similarly, *P. brassicacearum* and *P. kilonensis* share 190 proteins classified into 14 different categories, mostly enzymes, transporters and transcription factors, but both also share certain categories not found in any other combination of genomes, including lipid biosynthesis proteins, antimicrobial resistance genes, phrenyltransferases and protein kinases (Fig. 4b). Finally, *P. ogarae* and *P. kilonensis* share 200 proteins classified into 12 different categories, being enzymes, transporters and secretion systems mainly represented. The secretion-system category is the most abundant one within the shared fraction of both *P. ogarae* and *P. kilonensis* genomes, being clearly absent or very reduced within the other two genome comparisons (Fig. 4b). Secretion systems are complex and diverse key elements used by bacteria in a variety of processes [77]. In plant-associated bacteria, type-III and type-VI secretion systems (T3SS and T6SS, respectively) can function in interbacterial competition to suppress phytopathogens [78, 79]. Specifically, *P. ogarae* and *P. kilonensis* share the T3SS-I and the T6SS-III secretion systems (File S8) previously reported in F113 [80]. The co-occurrence of secretion systems in both *P. ogarae* and *P. kilonensis* might indicate a similar niche exploitation, which differs from that of *P. brassicacearum*.

Regarding the protein fraction specific to each genome (i.e. not found in the other two genomes), *P. ogarae* contains 417 proteins, 306 in *P. kilonensis* and 463 in *P. brassicacearum*, representing 7.36, 5.8 and 8.1 % of their total protein content, respectively. Among the functional categories of the proteins specific to *P. ogarae*, bacterial motility is the predominant trait of this genome and is supported by the presence of an additional flagellar system (File S8) previously reported to be involved in effective plant root colonization [81]. In the case of *P. kilonensis*, among the different specific proteins, we identified proteins for cellulose biosynthesis (BcsABCGF), several polyketide biosynthesis proteins and a type-II toxin-antitoxin system (File S8). Finally, among the specific proteins of *P. brassicacearum*, enzymes, transporters and transcriptional factors are the main distinctive categories.

### Phenotypic differences between *P. ogarae*, *P. brassicacearum*, *P. kilonensis* and *P. thivervalensis*

Phenotypic differences between *P. ogarae* F113^T^ and RDP1, *P. kilonensis* DSM 13647^T^, *P. brassicacearum* DSM 13227^T^ together with isolates WCS365, NFM421 and Q8r1-96, and *P. thivervalensis* DSM 13194^T^, were evaluated using Biolog GEN III, API 20 NE and API 50 CH tests. The results are summarized in Table 1 and extended information can be found in File S9. Among the total of 163 tests performed (94 Biolog, 20 API 20 NE and 49 API 50 CH), only 38 (23.3 %) showed differences among the strains tested. This was not unexpected given that all strains belong to closely related species.

**Table 1.** Differential phenotypic characteristics of strain F113^T^ and their closest species type strains Strains: 1, *P. ogarae* F113^T^; 2, *P. ogarae* RDP1; 3, *P. kilonensis* DSM 13647^T^; 4*, P. brassicacearum* DSM 13227^T^; 5, *Pseudomonas* sp. WCS365, 6, *P. brassicacearum* NFM421; 7, *P. fluorescens* Q8r1-96; 8, *P. thivervalensis* DSM 13194^T^.

Characteristic	1	2	3	4	5	6	7	8
**Assimilation of (Biolog):**								
N-acetyl-glucosamine	−	−	−	+	−	+	+	−
d-fructose	−	−	+	+	−	+	+	+
Inosine	+	+	+	+	−	+	+	−
d-arabitol	+	+	+	+	−	+	−	−
d-aspartic acid	+	+	−	+	+	+	+	+
l-arginine	+	+	+	+	+	+	+	−
l-histidine	−	+	+	+	+	+	−	−
l-serine	−	−	−	+	−	+	−	−
Pectin	+	+	+	−	−	+	−	−
d-galacturonic acid	−	+	−	+	+	+	+	−
l-galacturonic acid lactone	−	−	−	+	+	+	+	−
d-glucuronic acid	−	+	−	+	+	+	+	−
Tween 40	+	+	−	+	−	+	+	+
Acetic acid	+	+	+	+	−	−	−	−
**Sensitivity to (Biolog):**								
pH 5	−	−	−	−	−	+	+	−
1 % Sodium lactate	−	−	−	+	−	+	+	+
Troleandomycin	−	−	−	−	−	−	−	+
Guanidine HCl	−	−	+	−	−	−	−	+
Niaproof 4	+	−	−	−	−	+	+	−
Potassium tellurite	+	+	+	+	−	+	+	+
Aztreonam	−	−	+	−	−	+	−	+
**Oxidation of (API 50 CH):**								
Glycerol	+	+	w^a^	+^a^	+	+	+	+
d-arabinose	w	w	−	−	−	−	−	−
d-ribose	+	w	w^a^	w^a^	+	+	w	w^a^
l-xylose	w	−	−	−	−	−	−	−
Inositol	+	+	w*	w*	+	+	w	+
d-mannitol	+	+	w*	+	+	+	+	+
d-sorbitol	+	+	w*	+	+	+	+	+
d-melibiose	+	+	w*	−	+	+	w	+*
d-saccharose (sucrose)	+	+	w*	−*	+	+	+	+
d-trehalose	+	+	+	w*	+	+	+	+
Gentiobiose	w	w	−	−	−	−	−	w
d-lyxose	+	−	−	−	+	−	−	−
d-arabitol	+	+	−	w	+	w	w	+
**API 20 NE tests:**								
Reduction of nitrates	+	+	w*	−	+	w	w	−
Arginine dihydrolase	−	−	−	+	+	+	+	+
Gelatin hydrolysis (protease)	+	+	+	+	−	+	+	+
N-acetyl-glucosamine assimilation	−	−	−	+	+	+	+	+

For assimilation and oxidation of carbon compounds: positive (+), negative (-), weakly positive (w). For sensitivity: insensitive (+, can grow in its presence), sensitive (-, cannot grow in its presence).

Result differs from the published by Sikorsky *et al*. [67].

Among the different phenotypic features, *P. ogarae* F113^T^ and RDP1 are the only ones that cannot use d-fructose as the carbon source while all the remaining type strains can. Similarly, they both can weakly use d-arabinose, while none of the other strains tested can. There are also phenotypic features that distinguish *P. ogarae* from the rest of the type strains. For instance, there are six phenotypic traits in which *P. ogarae* and *P. kilonensis* differ: assimilation of d-aspartic acid, Tween 40, sensitivity to Guanidine HCl and Aztreonam and oxidation of gentiobiose and d-arabitol (Table 1). Compared to *P. brassicacearum* type strain, there are also six differential phenotypic features: assimilation of N-acetyl-glucosamine, pectin, l-galacturonic acid lactone, sensitivity to 1 % sodium lactate, oxidation of d-melibiose and gentiobiose. Finally, compared to *P. thivervalensis* type strain, there are 11 phenotypic traits in which they differ: assimilation of inosine, d-arabitol, l-arginine, pectin, acetic acid, sensitivity to 1 % sodium lactate, troleandomycin, guanidine HCl and aztreonam and activity of arginine dihydrolase. Most of the results obtained in our study agree with the ones previously published for *P. kilonensis*, *P. brassicacearum* and *P. thivervalensis* [67] based on the oxidation of carbon compounds and the enzymatic tests of API strips. However, we found that *P. brassicacearum* type strain can oxidate glycerol and d-sucrose, and *P. thivervalensis* type strain can oxidize d-melibiose, conversely to the results previously reported [67].

**Fig. 6.**
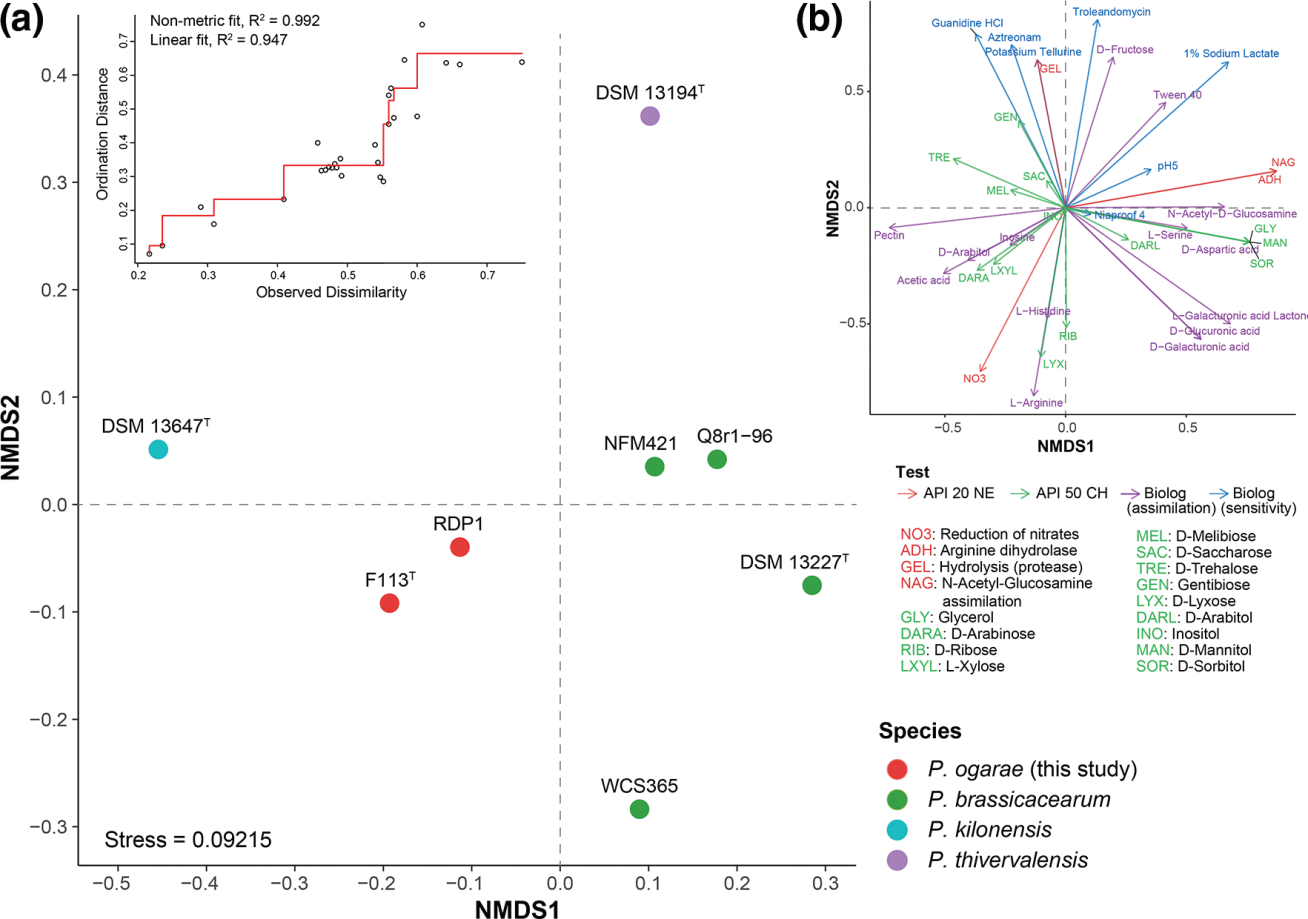
Non-metric multidimensional scaling (NMDS) ordination analysis of the distinctive phenotypic characters. (a) Scattered NMDS plot across NMDS1 and NMDS2 axes. Colours according to the species groups indicated in the legend. Shepard plot (upper left) indicating the statistics for goodness of fit between ordination distances and observed dissimilarity. (b) Intrinsic vectors driving the distribution pattern. Colour according to the test they belong to.

In addition, a non-metric multidimensional scaling (NMDS) ordination analysis was performed using the differential phenotypic traits among the strains analysed to visualize if the observed differences correspond to the species groups. As shown in Fig. 6, phenotypic traits clearly distinguish *P. kilonensis*, *P. thivervalensis*, *P. brassicacearum* and *P. ogarae* strains in separated observation groups. The variables that have the most effect in the distinction of *P. ogarae* are their ability to use acetic acid, d-arabitol and inosine as the carbon source, oxidation of d-arabinose and l-xylose and reduction of nitrates.

### Description of *Pseudomonas ogarae* sp. nov.

*Pseudomonas ogarae* (o.ga.rae. N.L. gen. n. *ogarae*, after Fergal O’Gara, Irish microbiologist who isolated the strain F113^T^ and defined its first plant growth-promoting features).

The cells of this species are aerobic, Gram-negative, non-spore-forming, motile by polar flagella and rod-shaped, being 0.5–1 µm wide and 1.5–5 µm in length. In minimal sucrose asparagine (SA) medium [82], colonies are yellowish with smooth and regular margins. After 24 h at 28 °C, they produce a yellow diffusible pigment giving a light yellow-green fluorescence when irradiated with UV light (254 nm). Results obtained with Biolog GEN III microplates indicate that cells can utilize as carbon and energy source the following substrates: d-trehalose, sucrose, α-d-glucose, d-mannose, d-galactose, inosine, d-sorbitol, d-mannitol, d-arabitol, myo-inositol, glycerol, d-aspartic acid, l-alanine, l-arginine, l-aspartic acid, l-glutamic acid, l-pyroglutamic acid, pectin, d-gluconic acid, mucic acid, quinic acid, d-saccharic acid, p-hydroxy-phenylacetic acid, methyl pyruvate, l-lactic acid, citric acid, α-keto-glutaric acid, l-malic acid, bromo-succinic acid, Tween 40, γ-amino-butyric acid, β-hydroxy-d,l-butyric acid and acetic acid. All other substrates included in Biolog GEN III were not utilized. Based on Biolog GEN III sensitivity assays, cells of this species can grow at pH 6 and 1% NaCl and resist rifamycin SV, lincomycin, niaproof 4, vancomycin and potassium tellurite. All other compounds included in Biolog GEN III sensitivity assays inhibited cell growth. Results obtained with API 50 CH strips indicate that cells oxidize the following substrates: glycerol, l-arabinose, d-ribose, d-xylose, d-galactose, d-glucose, d-fructose, d-mannose, inositol, d-mannitol, d-sorbitol, aesculin ferric citrate, d-melibiose, d-sucrose, d-trehalose, d-lyxose, d-fucose and d-arabitol, and weakly d-arabinose, d-xylose and gentiobiose. All other substrates included in the API 50 CH strips were not utilized. Results obtained with API 20 NE strips indicate that cells can utilize the following substrates as the carbon and energy source: glucose, arabinose, mannose, mannitol, potassium gluconate, capric acid, malate and trisodium citrate. All other substrates included in the API 20 NE strips were not utilized. In addition, the following enzyme activities were present in cells: hydrolysis (beta-glucosidase), hydrolysis (protease) and reduction of nitrates.

The type strain is strain F113^T^ (=DSM 112162^T^=CECT 30235^T^), isolated in 1992 from the rhizosphere of sugar beet.

## Conclusions

The results presented in this work substantiate the description of *P. ogarae* as a novel species, composed of strains F113^T^ (formerly *P. fluorescens* F113), RDP1, FR1, Ea_RS28, Ep_R1, ACN4, ACN7 and 1855–344. The differences compared to their closest species exist at the genomic, functional and phenotypic level. In addition, aside from the five previously described species of the *P. corrugata* subgroup, together with the proposal of *P. ogarae* presented here, there still remain 23 clusters of genomes that likely correspond to novel species that should be properly validated. Our results also evidence that the LPQ island of pathogenicity follows a clear distribution in species clusters and is present in almost half of the genomes analysed. Nevertheless, none of the closely related *P. ogarae*, *P. kilonensis* and *P. brassicacearum* genomes harbour this island and therefore could be the preferred agents within this subgroup for biotechnological applications.

## Supplementary Data

Supplementary material 1

Supplementary material 2
