# Supplementary material for: Comparative genomics of the Pseudomonas corrugata subgroup reveals high species diversity and allows the description of Pseudomonas ogarae sp. nov
Source: Microb Genom. 2021 Jun 29;7(6):000593. doi: 10.1099/mgen.0.000593 (PMC8461476; doi:10.1099/mgen.0.000593)
Supplement: Supplementary material 1 [file mgen-7-0593-s001.pdf]

Supplementary File S4 – page 2

Supplementary File S6 – page 36

Supplementary File S7 – page 43

Supplementary File S9 – page 45

Supplementary File S4. Whole genome-based taxonomic analyses of the 29 species-level clusters identified throughout the Type (Strain) Genome Server (TYGS).

Methods

Genomes within each species-level cluster identified in this work were separately submitted to the TYGS (Meier-Kolthoff and Göker, 2019). This is due to a limitation in the number of reference genomes currently accepted by the server. All settings and parameters were as default and previously described elsewhere (Liu et al., 2015). In brief, uploaded genomes were compared against all type strain genomes available in the TYGS via de MASH algorithm (Ondov et al., 2016). The ten type strains with the smallest MASH distances were selected. In addition, the 16S rDNA gene sequences of the uploaded genomes were extracted using RNAmmer (Lagesen et al., 2007) and blasted against the 16S rDNA gene sequences of 12,023 type strains currently included in the TYGS database to find the best 50 matching type strains according to bitscore, followed by a precise distance calculation using the GBDP approach under the 'coverage' algorithm and formula d_5 (Meier-Kolthoff et al., 2013). These distances were used to determine the 10 closest type strain genomes and further compared using GBDP and intergenomic distances, inferred under the 'trimming' algorithm and the d_5 formula (Meier-Kolthoff et al., 2013), using 100 replicates. Digital DDH values were calculated as described above. Minimum evolution tree was constructed with the resulting intergenomic distances, with 100 pseudo-bootstrap replicates branch support via FASTME v2.1.4 including SPR postprocessing (Lefort et al., 2015). Trees were visualized with PhyD3 (Kreft et al., 2017). Digital DDH clustering using a 70% radius around the 10 type strains was done as previously reported (Liu et al., 2015).

Results

For a concise description of each of the 29 species clusters identified, most of which are yet to be named (i.e., they do not contain any type strain), they were designated from 1 to 29, being 29 *P. brassicacearum* and 1 *P. mediterranea* according to Fig. 1 and throughout the text. The results per species cluster are shown below and include a table with the species clusters identified among the analyzed genomes (light red) and the closest type strains.

Species cluster 29 (*P. brassicacearum*, 19 genomes)

P. brassicacearum strains LBUM300, L13-6-12, PA1G7, NFM421, 51MFCVI2.1, 3Re2-7, LMG 21623, PP1_210F, **CCUG 51508 ^{T}**, 93F8, TM1A3, Delaware and Wood1, misclassified *P. fluorescens* strains AG1429, DSM 8569, Q8r1-96 and Wood1R, and unclassified isolates URIL14HWK12:I7 and WCS365.

Species cluster 29 (*P. brassicacearum*). Clusters identified

Kind	Species cluster	Subspecies cluster	Preferred name	Deposit	Authority
type strain	1	15	Pseudomonas brassicacearum	LMG 21623	Achouak et al. 2000 emend. Ivanova et al. 2009
user strain	1	15	Pseudomonas brassicacearum 3Re2-7	-	-
user strain	1	15	Pseudomonas brassicacearum 51MFCVI2 1	-	-
user strain	1	15	Pseudomonas brassicacearum 93F8	-	-
user strain	1	15	Pseudomonas brassicacearum Delaware	-	-
user strain	1	15	Pseudomonas brassicacearum L13-6-12	-	-
user strain	1	15	Pseudomonas brassicacearum LBUM300	-	-
user strain	1	15	Pseudomonas brassicacearum LMG 21623	-	-
user strain	1	15	Pseudomonas brassicacearum PA1G7	-	-
user strain	1	15	Pseudomonas brassicacearum PP1 210F	-	-
user strain	1	15	Pseudomonas brassicacearum subsp brassicacearum CCUG 51508 T	-	-
user strain	1	15	Pseudomonas brassicacearum subsp brassicacearum NFM421	-	-
user strain	1	15	Pseudomonas brassicacearum TM1A3	-	-
user strain	1	15	Pseudomonas brassicacearum Wood1	-	-
user strain	1	15	Pseudomonas fluorescens AG1429	-	-
user strain	1	15	Pseudomonas fluorescens DSM 8569	-	-
user strain	1	15	Pseudomonas fluorescens Q8r1-96	-	-
user strain	1	15	Pseudomonas fluorescens Wood1R	-	-
user strain	1	15	Pseudomonas sp URIL14HWK12 I7	-	-
user strain	1	15	Pseudomonas sp WCS365	-	-
type strain	2	0	Pseudomonas chlororaphis subsp. aurantiaca	DSM 19603	(Nakhimovskaya 1948) Peix et al. 2007
type strain	2	6	Pseudomonas chlororaphis subsp. aureofaciens	NBRC 3521	(Kluyver 1956) Peix et al. 2007
type strain	3	1	Pseudomonas silesiensis	A3	Kaminski et al. 2018
type strain	4	2	Pseudomonas frederiksbergensis	LMG 19851	Andersen et al. 2000
type strain	5	3	Pseudomonas migulae	NBRC 103157	Verhille et al. 1999
type strain	6	4	Pseudomonas thivervalensis	LMG 21626	Achouak et al. 2000
type strain	7	5	Pseudomonas mediterranea	CFBP 5447	Catara et al. 2002
type strain	8	7	Pseudomonas granadensis	LMG 27940	Pascual et al. 2015
type strain	9	8	Pseudomonas kilonensis	DSM 13647	Sikorski et al. 2001
type strain	10	9	Pseudomonas chlororaphis	LMG 5004	(Guignard and Sauvageau 1894) Bergey et al. 1930 emend. Peix et al. 2007
type strain	11	10	Pseudomonas corrugata	NCPPB 2445	Roberts and Scarlett 1981 emend. Sutra et al. 1997
type strain	11	10	Pseudomonas corrugata	DSM 7228	Roberts and Scarlett 1981 emend. Sutra et al. 1997
type strain	12	11	Pseudomonas taetrolens	DSM 21104	Haynes 1957
type strain	13	12	Pseudomonas lini	DSM 16768	Delorme et al. 2002
type strain	14	13	Pseudomonas chlororaphis subsp. piscium	DSM 21509	Burr et al. 2010
type strain	15	14	Pseudomonas haemolytica	DSM 108987T	Hofmann et al. 2020

Species cluster 28 (unnamed, 1 genome)

Pseudomonas sp. Root401.

Species cluster 28. Clusters identified

Kind	Species cluster	Subspecies cluster	Preferred name	Deposit	Authority
user strain	12	12	Pseudomonas sp Root401	-	-
type strain	11	11	Pseudomonas chlororaphis subsp. piscium	DSM 21509	Burr et al. 2010
type strain	10	10	Pseudomonas lini	DSM 16768	Delorme et al. 2002
type strain	9	9	Pseudomonas corrugata	NCPPB 2445	Roberts and Scarlett 1981 emend. Sutra et al. 1997
type strain	9	9	Pseudomonas corrugata	DSM 7228	Roberts and Scarlett 1981 emend. Sutra et al. 1997
type strain	8	8	Pseudomonas chlororaphis	LMG 5004	(Guignard and Sauvageau 1894) Bergey et al. 1930 emend. Peix et al. 2007
type strain	7	7	Pseudomonas kilonensis	DSM 13647	Sikorski et al. 2001
type strain	6	5	Pseudomonas mediterranea	CFBP 5447	Catara et al. 2002
type strain	5	4	Pseudomonas thivervalensis	LMG 21626	Achouak et al. 2000
type strain	4	3	Pseudomonas migulae	NBRC 103157	Verhille et al. 1999
type strain	3	2	Pseudomonas frederiksbergensis	LMG 19851	Andersen et al. 2000
type strain	2	1	Pseudomonas chlororaphis subsp. aurantiaca	DSM 19603	(Nakhimovskaya 1948) Peix et al. 2007
type strain	2	6	Pseudomonas chlororaphis subsp. aureofaciens	NBRC 3521	(Kluyver 1956) Peix et al. 2007
type strain	1	0	Pseudomonas brassicacearum	LMG 21623	Achouak et al. 2000 emend. Ivanova et al. 2009

Species cluster 27 (*P. kilonensis*, 6 genomes)

P. kilonensis strains **DSM 13647** ^{T}, BS3780, P12, S12 and unclassified isolates CFII68 and A25(2017).

Species cluster 27 (*P. kilonensis*). Clusters identified

Kind	Species cluster	Subspecies cluster	Preferred name	Deposit	Authority
type strain	8	14	Pseudomonas kilonensis	DSM 13647	Sikorski et al. 2001
user strain	8	14	Pseudomonas kilonensis BS3780	-	-
user strain	8	14	Pseudomonas kilonensis DSM 13647 T	-	-
user strain	8	14	Pseudomonas kilonensis P12	-	-
user strain	8	14	Pseudomonas sp A25 2017	-	-
user strain	8	14	Pseudomonas sp CFII68	-	-
user strain	8	13	Pseudomonas kilonensis S12	-	-
type strain	13	12	Pseudomonas haemolytica	DSM 108987T	Hofmann et al. 2020
type strain	12	11	Pseudomonas chlororaphis subsp. piscium	DSM 21509	Burr et al. 2010
type strain	11	10	Pseudomonas lini	DSM 16768	Delorme et al. 2002
type strain	9	9	Pseudomonas corrugata	NCPPB 2445	Roberts and Scarlett 1981 emend. Sutra et al. 1997
type strain	9	9	Pseudomonas corrugata	DSM 7228	Roberts and Scarlett 1981 emend. Sutra et al. 1997
type strain	10	8	Pseudomonas chlororaphis	LMG 5004	(Guignard and Sauvageau 1894) Bergey et al. 1930 emend. Peix et al. 2007
type strain	7	7	Pseudomonas arsenicoxydans	CECT 7543	Campos et al. 2011
type strain	6	6	Pseudomonas granadensis	LMG 27940	Pascual et al. 2015
type strain	2	5	Pseudomonas chlororaphis subsp. aureofaciens	NBRC 3521	(Kluyver 1956) Peix et al. 2007
type strain	5	4	Pseudomonas protegens	CHA0	Ramette et al. 2012
type strain	4	3	Pseudomonas mediterranea	CFBP 5447	Catara et al. 2002
type strain	3	2	Pseudomonas thivervalensis	LMG 21626	Achouak et al. 2000
type strain	2	1	Pseudomonas chlororaphis subsp. aurantiaca	DSM 19603	(Nakhimovskaya 1948) Peix et al. 2007
type strain	1	0	Pseudomonas brassicacearum	LMG 21623	Achouak et al. 2000 emend. Ivanova et al. 2009

Species cluster 26 (*P. ogarae*, this study, 8 genomes)

Misclassified *P. fluorescens* strains F113 and FR1, misclassified *P. kilonensis* strains ACN4, ACN7 and 1855-344, and unclassified isolates Ea_RS28, Ep_R1 and RDP1.

Species cluster 26. Clusters identified

Kind	Species cluster	Subspecies cluster	Preferred name	Deposit	Authority
user strain	21	21	Pseudomonas kilonensis 1855-344	-	-
user strain	21	21	Pseudomonas kilonensis ACN4	-	-
user strain	21	21	Pseudomonas kilonensis ACN7	-	-
user strain	21	21	Pseudomonas sp Ea RS28	-	-
user strain	21	21	Pseudomonas sp Ep R1	-	-
user strain	21	22	Pseudomonas fluorescens F113	-	-
user strain	21	22	Pseudomonas fluorescens FR1	-	-
user strain	21	22	Pseudomonas sp RDP1	-	-
type strain	20	20	Pseudomonas haemolytica	DSM 108987T	Hofmann et al. 2020
type strain	19	19	Pseudomonas mohnii	DSM 18327	Cámara et al. 2007
type strain	18	18	Pseudomonas chlororaphis subsp. piscium	DSM 21509	Burr et al. 2010
type strain	17	17	Pseudomonas lini	DSM 16768	Delorme et al. 2002
type strain	16	16	Pseudomonas taetrolens	DSM 21104	Haynes 1957
type strain	15	15	Pseudomonas prosekii	LMG 26867	Kosina et al. 2014
type strain	14	13	Pseudomonas chlororaphis	LMG 5004	(Guignard and Sauvageau 1894) Bergey et al. 1930 emend. Peix et al. 2007
type strain	13	14	Pseudomonas corrugata	NCPPB 2445	Roberts and Scarlett 1981 emend. Sutra et al. 1997
type strain	13	14	Pseudomonas corrugata	DSM 7228	Roberts and Scarlett 1981 emend. Sutra et al. 1997
type strain	12	12	Pseudomonas kilonensis	DSM 13647	Sikorski et al. 2001
type strain	11	11	Pseudomonas arsenicoxydans	CECT 7543	Campos et al. 2011
type strain	10	10	Pseudomonas granadensis	LMG 27940	Pascual et al. 2015
type strain	9	9	Pseudomonas trivialis	LMG 21464	Behrendt et al. 2003
type strain	8	7	Pseudomonas mediterranea	CFBP 5447	Catara et al. 2002
type strain	7	6	Pseudomonas thivervalensis	LMG 21626	Achouak et al. 2000
type strain	6	5	Pseudomonas migulae	NBRC 103157	Verhille et al. 1999
type strain	5	4	Pseudomonas laurylsulfativorans	AP3_22	Furmanczyk et al. 2019
type strain	4	3	Pseudomonas laurylsulfatiphila	AP3_16	Furmanczyk et al. 2019
type strain	3	2	Pseudomonas chlororaphis subsp. aurantiaca	DSM 19603	(Nakhimovskaya 1948) Peix et al. 2007
type strain	3	8	Pseudomonas chlororaphis subsp. aureofaciens	NBRC 3521	(Kluyver 1956) Peix et al. 2007
type strain	2	1	Pseudomonas brassicacearum	LMG 21623	Achouak et al. 2000 emend. Ivanova et al. 2009
type strain	1	0	Pseudomonas caspiana	FBF102	Busquets et al. 2017

Species cluster 25 (unnamed, 3 genomes)

Misclassified *P. kilonensis* strain ZKA7, misclassified *P. brassiacearum* strain S-1 and misclassified *P. fluorescens* strain et76.

Species cluster 25. Clusters identified

Kind	Species cluster	Subspecies cluster	Preferred name	Deposit	Authority
user strain	12	12	Pseudomonas brassicacearum S-1	-	-
user strain	12	12	Pseudomonas fluorescens et76	-	-
user strain	12	12	Pseudomonas kilonensis ZKA7	-	-
type strain	11	11	Pseudomonas haemolytica	DSM 108987T	Hofmann et al. 2020
type strain	10	10	Pseudomonas chlororaphis subsp. piscium	DSM 21509	Burr et al. 2010
type strain	9	9	Pseudomonas lini	DSM 16768	Delorme et al. 2002
type strain	8	8	Pseudomonas corrugata	NCPPB 2445	Roberts and Scarlett 1981 emend. Sutra et al. 1997
type strain	8	8	Pseudomonas corrugata	DSM 7228	Roberts and Scarlett 1981 emend. Sutra et al. 1997
type strain	7	7	Pseudomonas chlororaphis	LMG 5004	(Guignard and Sauvageau 1894) Bergey et al. 1930 emend. Peix et al. 2007
type strain	6	6	Pseudomonas kilonensis	DSM 13647	Sikorski et al. 2001
type strain	5	4	Pseudomonas mediterranea	CFBP 5447	Catara et al. 2002
type strain	4	3	Pseudomonas thivervalensis	LMG 21626	Achouak et al. 2000
type strain	3	2	Pseudomonas migulae	NBRC 103157	Verhille et al. 1999
type strain	2	1	Pseudomonas chlororaphis subsp. aurantiaca	DSM 19603	(Nakhimovskaya 1948) Peix et al. 2007
type strain	2	5	Pseudomonas chlororaphis subsp. aureofaciens	NBRC 3521	(Kluyver 1956) Peix et al. 2007
type strain	1	0	Pseudomonas brassicacearum	LMG 21623	Achouak et al. 2000 emend. Ivanova et al. 2009

Species cluster 24 (unnamed, 13 genomes)

Unclassified isolates NFACC51, NFACC48-1, NFACC54, NFACC44-2, NFACC50-1, NFACC07-1, NFACC46-3, NFACC05-1, NFACC08-1, NFACC17-2, NFACC23-1, NFACC16-2 and misclasified *P. fluorescens* strain FW300-N2E2.

Species cluster 24. Clusters identified

Kind	Species cluster	Subspecies cluster	Preferred name	Deposit	Authority
user strain	15	15	Pseudomonas sp NFACC16-2	-	-
user strain	15	15	Pseudomonas sp NFACC17-2	-	-
user strain	15	15	Pseudomonas sp NFACC23-1	-	-
user strain	15	16	Pseudomonas fluorescens FW300-N2E2	-	-
user strain	15	16	Pseudomonas sp NFACC05-1	-	-
user strain	15	16	Pseudomonas sp NFACC07-1	-	-
user strain	15	16	Pseudomonas sp NFACC08-1	-	-
user strain	15	16	Pseudomonas sp NFACC44-2	-	-
user strain	15	16	Pseudomonas sp NFACC46-3	-	-
user strain	15	16	Pseudomonas sp NFACC48-1	-	-
user strain	15	16	Pseudomonas sp NFACC50-1	-	-
user strain	15	16	Pseudomonas sp NFACC51	-	-
user strain	15	16	Pseudomonas sp NFACC54	-	-
type strain	14	14	Pseudomonas haemolytica	DSM 108987T	Hofmann et al. 2020
type strain	13	13	Pseudomonas chlororaphis subsp. piscium	DSM 21509	Burr et al. 2010
type strain	12	12	Pseudomonas lini	DSM 16768	Delorme et al. 2002
type strain	11	11	Pseudomonas prosekii	LMG 26867	Kosina et al. 2014
type strain	10	9	Pseudomonas chlororaphis	LMG 5004	(Guignard and Sauvageau 1894) Bergey et al. 1930 emend. Peix et al. 2007
type strain	9	10	Pseudomonas corrugata	NCPPB 2445	Roberts and Scarlett 1981 emend. Sutra et al. 1997
type strain	9	10	Pseudomonas corrugata	DSM 7228	Roberts and Scarlett 1981 emend. Sutra et al. 1997
type strain	8	8	Pseudomonas kilonensis	DSM 13647	Sikorski et al. 2001
type strain	7	7	Pseudomonas arsenicoxydans	CECT 7543	Campos et al. 2011
type strain	6	6	Pseudomonas granadensis	LMG 27940	Pascual et al. 2015
type strain	5	4	Pseudomonas mediterranea	CFBP 5447	Catara et al. 2002
type strain	4	3	Pseudomonas thivervalensis	LMG 21626	Achouak et al. 2000
type strain	3	2	Pseudomonas migulae	NBRC 103157	Verhille et al. 1999
type strain	2	1	Pseudomonas chlororaphis subsp. aurantiaca	DSM 19603	(Nakhimovskaya 1948) Peix et al. 2007
type strain	2	5	Pseudomonas chlororaphis subsp. aureofaciens	NBRC 3521	(Kluyver 1956) Peix et al. 2007
type strain	1	0	Pseudomonas brassicacearum	LMG 21623	Achouak et al. 2000 emend. Ivanova et al. 2009

Species cluster 23 (unnamed, 8 genomes)

Unclassified isolates NFIX10, NFACC06-1, NFACC11-2, NFACC56-3, NFACC52, NFACC14, NFACC15-1 and NFACC13-1.

Species cluster 23. Clusters identified

Kind	Species cluster	Subspecies cluster	Preferred name	Deposit	Authority
user strain	14	14	Pseudomonas sp NFACC13-1	-	-
user strain	14	14	Pseudomonas sp NFACC14	-	-
user strain	14	14	Pseudomonas sp NFACC15-1	-	-
user strain	14	15	Pseudomonas sp NFACC06-1	-	-
user strain	14	15	Pseudomonas sp NFACC11-2	-	-
user strain	14	15	Pseudomonas sp NFACC52	-	-
user strain	14	15	Pseudomonas sp NFACC56-3	-	-
user strain	14	15	Pseudomonas sp NFIX10	-	-
type strain	13	13	Pseudomonas chlororaphis subsp. piscium	DSM 21509	Burr et al. 2010
type strain	12	12	Pseudomonas lini	DSM 16768	Delorme et al. 2002
type strain	11	11	Pseudomonas prosekii	LMG 26867	Kosina et al. 2014
type strain	10	9	Pseudomonas chlororaphis	LMG 5004	(Guignard and Sauvageau 1894) Bergey et al. 1930 emend. Peix et al. 2007
type strain	9	10	Pseudomonas corrugata	NCPPB 2445	Roberts and Scarlett 1981 emend. Sutra et al. 1997
type strain	9	10	Pseudomonas corrugata	DSM 7228	Roberts and Scarlett 1981 emend. Sutra et al. 1997
type strain	8	8	Pseudomonas kilonensis	DSM 13647	Sikorski et al. 2001
type strain	7	7	Pseudomonas granadensis	LMG 27940	Pascual et al. 2015
type strain	6	5	Pseudomonas mediterranea	CFBP 5447	Catara et al. 2002
type strain	5	4	Pseudomonas thivervalensis	LMG 21626	Achouak et al. 2000
type strain	4	3	Pseudomonas migulae	NBRC 103157	Verhille et al. 1999
type strain	3	2	Pseudomonas chlororaphis subsp. aurantiaca	DSM 19603	(Nakhimovskaya 1948) Peix et al. 2007
type strain	3	6	Pseudomonas chlororaphis subsp. aureofaciens	NBRC 3521	(Kluyver 1956) Peix et al. 2007
type strain	2	1	Pseudomonas brassicacearum	LMG 21623	Achouak et al. 2000 emend. Ivanova et al. 2009
type strain	1	0	Pseudomonas atagosis	PS14	Morimoto et al. 2020

Species cluster 22 (unnamed, 2 genomes)

Misclassified *P. fluorescens* strain FW300-N2C3 and unclassified isolate MPBD7-1.

Species cluster 22. Clusters identified

Kind	Species cluster	Subspecies cluster	Preferred name	Deposit	Authority
user strain	12	12	Pseudomonas fluorescens FW300-N2C3	-	-
user strain	12	12	Pseudomonas sp MPBD7-1	-	-
type strain	11	11	Pseudomonas chlororaphis subsp. piscium	DSM 21509	Burr et al. 2010
type strain	10	10	Pseudomonas lini	DSM 16768	Delorme et al. 2002
type strain	9	9	Pseudomonas corrugata	NCPPB 2445	Roberts and Scarlett 1981 emend. Sutra et al. 1997
type strain	9	9	Pseudomonas corrugata	DSM 7228	Roberts and Scarlett 1981 emend. Sutra et al. 1997
type strain	8	8	Pseudomonas chlororaphis	LMG 5004	(Guignard and Sauvageau 1894) Bergey et al. 1930 emend. Peix et al. 2007
type strain	7	7	Pseudomonas kilonensis	DSM 13647	Sikorski et al. 2001
type strain	6	5	Pseudomonas mediterranea	CFBP 5447	Catara et al. 2002
type strain	5	4	Pseudomonas thivervalensis	LMG 21626	Achouak et al. 2000
type strain	4	3	Pseudomonas migulae	NBRC 103157	Verhille et al. 1999
type strain	3	2	Pseudomonas frederiksbergensis	LMG 19851	Andersen et al. 2000
type strain	2	1	Pseudomonas chlororaphis subsp. aurantiaca	DSM 19603	(Nakhimovskaya 1948) Peix et al. 2007
type strain	2	6	Pseudomonas chlororaphis subsp. aureofaciens	NBRC 3521	(Kluyver 1956) Peix et al. 2007
type strain	1	0	Pseudomonas brassicacearum	LMG 21623	Achouak et al. 2000 emend. Ivanova et al. 2009

Species cluster 21 (unnamed, 2 genomes)

Unclassified isolates NFACC24-1 and NFACC37-1.

Species cluster 21. Clusters identified

Kind	Species cluster	Subspecies cluster	Preferred name	Deposit	Authority
user strain	13	13	Pseudomonas sp NFACC24-1	-	-
user strain	13	13	Pseudomonas sp NFACC37-1	-	-
type strain	12	12	Pseudomonas chlororaphis subsp. piscium	DSM 21509	Burr et al. 2010
type strain	11	11	Pseudomonas lini	DSM 16768	Delorme et al. 2002
type strain	10	10	Pseudomonas prosekii	LMG 26867	Kosina et al. 2014
type strain	9	8	Pseudomonas chlororaphis	LMG 5004	(Guignard and Sauvageau 1894) Bergey et al. 1930 emend. Peix et al. 2007
type strain	8	9	Pseudomonas corrugata	NCPPB 2445	Roberts and Scarlett 1981 emend. Sutra et al. 1997
type strain	8	9	Pseudomonas corrugata	DSM 7228	Roberts and Scarlett 1981 emend. Sutra et al. 1997
type strain	7	7	Pseudomonas kilonensis	DSM 13647	Sikorski et al. 2001
type strain	6	6	Pseudomonas arsenicoxydans	CECT 7543	Campos et al. 2011
type strain	5	4	Pseudomonas mediterranea	CFBP 5447	Catara et al. 2002
type strain	4	3	Pseudomonas thivervalensis	LMG 21626	Achouak et al. 2000
type strain	3	2	Pseudomonas migulae	NBRC 103157	Verhille et al. 1999
type strain	2	1	Pseudomonas chlororaphis subsp. aurantiaca	DSM 19603	(Nakhimovskaya 1948) Peix et al. 2007
type strain	2	5	Pseudomonas chlororaphis subsp. aureofaciens	NBRC 3521	(Kluyver 1956) Peix et al. 2007
type strain	1	0	Pseudomonas brassicacearum	LMG 21623	Achouak et al. 2000 emend. Ivanova et al. 2009

Species cluster 20 (unnamed, 2 genomes)

Misclassified *P. fluorescens* strain FW300_25_6 and unclassified isolate NFACC04-2.

Species cluster 20. Clusters identified

Kind	Species cluster	Subspecies cluster	Preferred name	Deposit	Authority
user strain	15	15	Pseudomonas fluorescens FW300 25 6	-	-
user strain	15	15	Pseudomonas sp NFACC04-2	-	-
type strain	14	14	Pseudomonas haemolytica	DSM 108987T	Hofmann et al. 2020
type strain	13	13	Pseudomonas chlororaphis subsp. piscium	DSM 21509	Burr et al. 2010
type strain	12	12	Pseudomonas lini	DSM 16768	Delorme et al. 2002
type strain	11	11	Pseudomonas prosekii	LMG 26867	Kosina et al. 2014
type strain	10	10	Pseudomonas corrugata	NCPPB 2445	Roberts and Scarlett 1981 emend. Sutra et al. 1997
type strain	10	10	Pseudomonas corrugata	DSM 7228	Roberts and Scarlett 1981 emend. Sutra et al. 1997
type strain	9	9	Pseudomonas kilonensis	DSM 13647	Sikorski et al. 2001
type strain	8	8	Pseudomonas arsenicoxydans	CECT 7543	Campos et al. 2011
type strain	7	7	Pseudomonas granadensis	LMG 27940	Pascual et al. 2015
type strain	6	5	Pseudomonas protegens	CHA0	Ramette et al. 2012
type strain	5	4	Pseudomonas mediterranea	CFBP 5447	Catara et al. 2002
type strain	4	3	Pseudomonas thivervalensis	LMG 21626	Achouak et al. 2000
type strain	3	2	Pseudomonas migulae	NBRC 103157	Verhille et al. 1999
type strain	2	1	Pseudomonas chlororaphis subsp. aurantiaca	DSM 19603	(Nakhimovskaya 1948) Peix et al. 2007
type strain	2	6	Pseudomonas chlororaphis subsp. aureofaciens	NBRC 3521	(Kluyver 1956) Peix et al. 2007
type strain	1	0	Pseudomonas brassicacearum	LMG 21623	Achouak et al. 2000 emend. Ivanova et al. 2009

Species cluster 19 (*P. thivervalensis*, 3 genomes)

P. thivervalensis strains LMG 21626, **DSM 12194** [T] and PITR2.

Species cluster 19 (*P. thivervalensis*). Clusters identified

Kind	Species cluster	Subspecies cluster	Preferred name	Deposit	Authority
type strain	4	14	Pseudomonas thivervalensis	LMG 21626	Achouak et al. 2000
user strain	4	14	Pseudomonas thivervalensis DSM 13194	-	-
user strain	4	14	Pseudomonas thivervalensis LMG 21626	-	-
user strain	4	14	Pseudomonas thivervalensis PITR2	-	-
type strain	14	13	Pseudomonas haemolytica	DSM 108987T	Hofmann et al. 2020
type strain	13	12	Pseudomonas chlororaphis subsp. piscium	DSM 21509	Burr et al. 2010
type strain	12	11	Pseudomonas lini	DSM 16768	Delorme et al. 2002
type strain	11	10	Pseudomonas prosekii	LMG 26867	Kosina et al. 2014
type strain	10	9	Pseudomonas corrugata	NCPPB 2445	Roberts and Scarlett 1981 emend. Sutra et al. 1997
type strain	10	9	Pseudomonas corrugata	DSM 7228	Roberts and Scarlett 1981 emend. Sutra et al. 1997
type strain	9	8	Pseudomonas chlororaphis	LMG 5004	(Guignard and Sauvageau 1894) Bergey et al. 1930 emend. Peix et al. 2007
type strain	8	7	Pseudomonas kilonensis	DSM 13647	Sikorski et al. 2001
type strain	7	6	Pseudomonas arsenicoxydans	CECT 7543	Campos et al. 2011
type strain	6	5	Pseudomonas yamanorum	LMG 27247	Arnau et al. 2015
type strain	3	4	Pseudomonas chlororaphis subsp. aureofaciens	NBRC 3521	(Kluyver 1956) Peix et al. 2007
type strain	5	3	Pseudomonas mediterranea	CFBP 5447	Catara et al. 2002
type strain	3	2	Pseudomonas chlororaphis subsp. aurantiaca	DSM 19603	(Nakhimovskaya 1948) Peix et al. 2007
type strain	2	1	Pseudomonas brassicacearum	LMG 21623	Achouak et al. 2000 emend. Ivanova et al. 2009
type strain	1	0	Pseudomonas atagosis	PS14	Morimoto et al. 2020

Species cluster 18 (unnamed, 2 genomes)

Misclassified *P. fluorescens* strain Pf275 and unclassified isolate 43mfcvi1.1.

Species cluster 18. Clusters identified

Kind	Species cluster	Subspecies cluster	Preferred name	Deposit	Authority
user strain	16	16	Pseudomonas fluorescens Pf275	-	-
user strain	16	16	Pseudomonas sp 43mfcvi1 1	-	-
type strain	15	15	Pseudomonas mohnii	DSM 18327	Cámara et al. 2007
type strain	14	14	Pseudomonas chlororaphis subsp. piscium	DSM 21509	Burr et al. 2010
type strain	13	13	Pseudomonas lini	DSM 16768	Delorme et al. 2002
type strain	12	11	Pseudomonas chlororaphis	LMG 5004	(Guignard and Sauvageau 1894) Bergey et al. 1930 emend. Peix et al. 2007
type strain	11	12	Pseudomonas corrugata	NCPPB 2445	Roberts and Scarlett 1981 emend. Sutra et al. 1997
type strain	11	12	Pseudomonas corrugata	DSM 7228	Roberts and Scarlett 1981 emend. Sutra et al. 1997
type strain	10	10	Pseudomonas kilonensis	DSM 13647	Sikorski et al. 2001
type strain	9	8	Pseudomonas mediterranea	CFBP 5447	Catara et al. 2002
type strain	8	7	Pseudomonas thivervalensis	LMG 21626	Achouak et al. 2000
type strain	7	6	Pseudomonas migulae	NBRC 103157	Verhille et al. 1999
type strain	6	5	Pseudomonas frederiksbergensis	LMG 19851	Andersen et al. 2000
type strain	5	4	Pseudomonas cedrina	DSM 17516	Dabboussi et al. 2002 emend. Behrendt et al. 2009
type strain	4	3	Pseudomonas laurylsulfatiphila	AP3_16	Furmanczyk et al. 2019
type strain	3	2	Pseudomonas chlororaphis subsp. aurantiaca	DSM 19603	(Nakhimovskaya 1948) Peix et al. 2007
type strain	3	9	Pseudomonas chlororaphis subsp. aureofaciens	NBRC 3521	(Kluyver 1956) Peix et al. 2007
type strain	2	1	Pseudomonas brassicacearum	LMG 21623	Achouak et al. 2000 emend. Ivanova et al. 2009
type strain	1	0	Pseudomonas atagosis	PS14	Morimoto et al. 2020

Species cluster 17 (unnamed, 1 genome)

Misclassified *P. fluorescens* strain 2P24.

Species cluster 17. Clusters identified

Kind	Species cluster	Subspecies cluster	Preferred name	Deposit	Authority
user strain	12	12	Pseudomonas fluorescens 2P24	-	-
type strain	11	11	Pseudomonas chlororaphis subsp. piscium	DSM 21509	Burr et al. 2010
type strain	10	10	Pseudomonas lini	DSM 16768	Delorme et al. 2002
type strain	9	9	Pseudomonas prosekii	LMG 26867	Kosina et al. 2014
type strain	8	8	Pseudomonas corrugata	NCPPB 2445	Roberts and Scarlett 1981 emend. Sutra et al. 1997
type strain	8	8	Pseudomonas corrugata	DSM 7228	Roberts and Scarlett 1981 emend. Sutra et al. 1997
type strain	7	7	Pseudomonas chlororaphis	LMG 5004	(Guignard and Sauvageau 1894) Bergey et al. 1930 emend. Peix et al. 2007
type strain	6	6	Pseudomonas kilonensis	DSM 13647	Sikorski et al. 2001
type strain	5	4	Pseudomonas mediterranea	CFBP 5447	Catara et al. 2002
type strain	4	3	Pseudomonas thivervalensis	LMG 21626	Achouak et al. 2000
type strain	3	2	Pseudomonas migulae	NBRC 103157	Verhille et al. 1999
type strain	2	1	Pseudomonas chlororaphis subsp. aurantiaca	DSM 19603	(Nakhimovskaya 1948) Peix et al. 2007
type strain	2	5	Pseudomonas chlororaphis subsp. aureofaciens	NBRC 3521	(Kluyver 1956) Peix et al. 2007
type strain	1	0	Pseudomonas brassicacearum	LMG 21623	Achouak et al. 2000 emend. Ivanova et al. 2009

Species cluster 16 (unnamed, 2 genomes)

Misclassified *P. brassicacearum* strains 36D4 and 36B7.

Species cluster 16. Clusters identified

Kind	Species cluster	Subspecies cluster	Preferred name	Deposit	Authority
user strain	20	20	Pseudomonas brassicacearum 36B7	-	-
user strain	20	21	Pseudomonas brassicacearum 36D4	-	-
type strain	19	19	Pseudomonas chlororaphis subsp. piscium	DSM 21509	Burr et al. 2010
type strain	18	18	Pseudomonas lini	DSM 16768	Delorme et al. 2002
type strain	17	17	Pseudomonas prosekii	LMG 26867	Kosina et al. 2014
type strain	16	15	Pseudomonas chlororaphis	LMG 5004	(Guignard and Sauvageau 1894) Bergey et al. 1930 emend. Peix et al. 2007
type strain	15	16	Pseudomonas corrugata	NCPPB 2445	Roberts and Scarlett 1981 emend. Sutra et al. 1997
type strain	15	16	Pseudomonas corrugata	DSM 7228	Roberts and Scarlett 1981 emend. Sutra et al. 1997
type strain	14	14	Pseudomonas kilonensis	DSM 13647	Sikorski et al. 2001
type strain	13	13	Pseudomonas veronii	DSM 11331	Elomari et al. 1996
type strain	12	12	Pseudomonas arsenicoxydans	CECT 7543	Campos et al. 2011
type strain	11	11	Pseudomonas yamanorum	LMG 27247	Arnau et al. 2015
type strain	10	9	Pseudomonas protegens	CHA0	Ramette et al. 2012
type strain	9	8	Pseudomonas mediterranea	CFBP 5447	Catara et al. 2002
type strain	8	7	Pseudomonas thivervalensis	LMG 21626	Achouak et al. 2000
type strain	7	6	Pseudomonas lurida	LMG 21995	Behrendt et al. 2007
type strain	6	5	Pseudomonas migulae	NBRC 103157	Verhille et al. 1999
type strain	5	4	Pseudomonas palleroniana	LMG 23076	Gardan et al. 2002
type strain	4	3	Pseudomonas nabeulensis	E10BT	Oueslati et al. 2020
type strain	3	2	Pseudomonas chlororaphis subsp. aurantiaca	DSM 19603	(Nakhimovskaya 1948) Peix et al. 2007
type strain	3	10	Pseudomonas chlororaphis subsp. aureofaciens	NBRC 3521	(Kluyver 1956) Peix et al. 2007
type strain	2	1	Pseudomonas brassicacearum	LMG 21623	Achouak et al. 2000 emend. Ivanova et al. 2009
type strain	1	0	Pseudomonas tolaasii	NCPPB 2192	Paine 1919

Species cluster 15 (unnamed, 1 genome)

Misclassified *P. brassicacearum* strain DF41.

Species cluster 15. Clusters identified

Kind	Species cluster	Subspecies cluster	Preferred name	Deposit	Authority
user strain	19	18	Pseudomonas brassicacearum DF41	-	-
type strain	18	17	Pseudomonas chlororaphis subsp. piscium	DSM 21509	Burr et al. 2010
type strain	17	16	Pseudomonas lini	DSM 16768	Delorme et al. 2002
type strain	16	15	Pseudomonas prosekii	LMG 26867	Kosina et al. 2014
type strain	15	14	Pseudomonas corrugata	NCPPB 2445	Roberts and Scarlett 1981 emend. Sutra et al. 1997
type strain	15	14	Pseudomonas corrugata	DSM 7228	Roberts and Scarlett 1981 emend. Sutra et al. 1997
type strain	14	13	Pseudomonas chlororaphis	LMG 5004	(Guignard and Sauvageau 1894) Bergey et al. 1930 emend. Peix et al. 2007
type strain	13	12	Pseudomonas kilonensis	DSM 13647	Sikorski et al. 2001
type strain	12	11	Pseudomonas veronii	DSM 11331	Elomari et al. 1996
type strain	11	10	Pseudomonas arsenicoxydans	CECT 7543	Campos et al. 2011
type strain	10	9	Pseudomonas trivialis	LMG 21464	Behrendt et al. 2003
type strain	9	8	Pseudomonas mediterranea	CFBP 5447	Catara et al. 2002
type strain	8	7	Pseudomonas thivervalensis	LMG 21626	Achouak et al. 2000
type strain	7	6	Pseudomonas extremaustralis	14-3	López et al. 2010
type strain	6	5	Pseudomonas migulae	NBRC 103157	Verhille et al. 1999
type strain	5	4	Pseudomonas palleroniana	LMG 23076	Gardan et al. 2002
type strain	4	3	Pseudomonas antarctica	LMG 22709	Reddy et al. 2004
type strain	3	2	Pseudomonas nabeulensis	E10BT	Oueslati et al. 2020
type strain	2	1	Pseudomonas chlororaphis subsp. aurantiaca	DSM 19603	(Nakhimovskaya 1948) Peix et al. 2007
type strain	1	0	Pseudomonas brassicacearum	LMG 21623	Achouak et al. 2000 emend. Ivanova et al. 2009

Species cluster 14 (unnamed, 1 genome)

Unclassified isolate 11K1

Species cluster 14. Clusters identified

Kind	Species cluster	Subspecies cluster	Preferred name	Deposit	Authority
user strain	13	12	Pseudomonas sp 11K1	-	-
type strain	12	11	Pseudomonas chlororaphis subsp. piscium	DSM 21509	Burr et al. 2010
type strain	11	10	Pseudomonas lini	DSM 16768	Delorme et al. 2002
type strain	10	9	Pseudomonas prosekii	LMG 26867	Kosina et al. 2014
type strain	9	8	Pseudomonas corrugata	NCPPB 2445	Roberts and Scarlett 1981 emend. Sutra et al. 1997
type strain	9	8	Pseudomonas corrugata	DSM 7228	Roberts and Scarlett 1981 emend. Sutra et al. 1997
type strain	8	7	Pseudomonas chlororaphis	LMG 5004	(Guignard and Sauvageau 1894) Bergey et al. 1930 emend. Peix et al. 2007
type strain	7	6	Pseudomonas kilonensis	DSM 13647	Sikorski et al. 2001
type strain	6	5	Pseudomonas arsenicoxydans	CECT 7543	Campos et al. 2011
type strain	5	4	Pseudomonas mediterranea	CFBP 5447	Catara et al. 2002
type strain	4	3	Pseudomonas thivervalensis	LMG 21626	Achouak et al. 2000
type strain	3	2	Pseudomonas silesiensis	A3	Kaminski et al. 2018
type strain	2	1	Pseudomonas chlororaphis subsp. aurantiaca	DSM 19603	(Nakhimovskaya 1948) Peix et al. 2007
type strain	1	0	Pseudomonas brassicacearum	LMG 21623	Achouak et al. 2000 emend. Ivanova et al. 2009

Species cluster 13 (unnamed, 2 genomes)

Misclassified *P. fluorescens* strain Q2-87 and unclassified isolate Q12-87.

Species cluster 13. Clusters identified

Kind	Species cluster	Subspecies cluster	Preferred name	Deposit	Authority
user strain	14	13	Pseudomonas fluorescens Q2-87	-	-
user strain	14	13	Pseudomonas sp Q12-87	-	-
type strain	13	12	Pseudomonas haemolytica	DSM 108987T	Hofmann et al. 2020
type strain	12	11	Pseudomonas lini	DSM 16768	Delorme et al. 2002
type strain	11	9	Pseudomonas chlororaphis	LMG 5004	(Guignard and Sauvageau 1894) Bergey et al. 1930 emend. Peix et al. 2007
type strain	10	10	Pseudomonas corrugata	NCPPB 2445	Roberts and Scarlett 1981 emend. Sutra et al. 1997
type strain	10	10	Pseudomonas corrugata	DSM 7228	Roberts and Scarlett 1981 emend. Sutra et al. 1997
type strain	9	8	Pseudomonas kilonensis	DSM 13647	Sikorski et al. 2001
type strain	8	7	Pseudomonas mediterranea	CFBP 5447	Catara et al. 2002
type strain	7	6	Pseudomonas thivervalensis	LMG 21626	Achouak et al. 2000
type strain	6	5	Pseudomonas migulae	NBRC 103157	Verhille et al. 1999
type strain	5	4	Pseudomonas frederiksbergensis	LMG 19851	Andersen et al. 2000
type strain	4	3	Pseudomonas silesiensis	A3	Kaminski et al. 2018
type strain	3	2	Pseudomonas cedrina	DSM 17516	Dabboussi et al. 2002 emend. Behrendt et al. 2009
type strain	2	1	Pseudomonas chlororaphis subsp. aurantiaca	DSM 19603	(Nakhimovskaya 1948) Peix et al. 2007
type strain	1	0	Pseudomonas brassicacearum	LMG 21623	Achouak et al. 2000 emend. Ivanova et al. 2009

Species cluster 12 (unnamed, 5 genomes)

Misclassified *P. brassicacearum* strain LZ-4 and unclassified isolates Fig-3, S211(2017), A214 and PICF6.

Species cluster 12. Clusters identified

Kind	Species cluster	Subspecies cluster	Preferred name	Deposit	Authority
user strain	14	14	Pseudomonas brassicacearum LZ-4	-	-
user strain	14	14	Pseudomonas sp A214	-	-
user strain	14	14	Pseudomonas sp Fig-3	-	-
user strain	14	14	Pseudomonas sp PICF6	-	-
user strain	14	14	Pseudomonas sp s211 2017	-	-
type strain	13	13	Pseudomonas chlororaphis subsp. piscium	DSM 21509	Burr et al. 2010
type strain	12	12	Pseudomonas lini	DSM 16768	Delorme et al. 2002
type strain	11	10	Pseudomonas chlororaphis	LMG 5004	(Guignard and Sauvageau 1894) Bergey et al. 1930 emend. Peix et al. 2007
type strain	10	11	Pseudomonas corrugata	NCPPB 2445	Roberts and Scarlett 1981 emend. Sutra et al. 1997
type strain	10	11	Pseudomonas corrugata	DSM 7228	Roberts and Scarlett 1981 emend. Sutra et al. 1997
type strain	9	9	Pseudomonas kilonensis	DSM 13647	Sikorski et al. 2001
type strain	8	8	Pseudomonas mandelii	LMG 21607	Verhille et al. 1999
type strain	7	7	Pseudomonas granadensis	LMG 27940	Pascual et al. 2015
type strain	6	6	Pseudomonas fluorescens	DSM 50090	Migula 1895
type strain	5	4	Pseudomonas mediterranea	CFBP 5447	Catara et al. 2002
type strain	4	3	Pseudomonas thivervalensis	LMG 21626	Achouak et al. 2000
type strain	3	2	Pseudomonas frederiksbergensis	LMG 19851	Andersen et al. 2000
type strain	2	1	Pseudomonas chlororaphis subsp. aurantiaca	DSM 19603	(Nakhimovskaya 1948) Peix et al. 2007
type strain	2	5	Pseudomonas chlororaphis subsp. aureofaciens	NBRC 3521	(Kluyver 1956) Peix et al. 2007
type strain	1	0	Pseudomonas brassicacearum	LMG 21623	Achouak et al. 2000 emend. Ivanova et al. 2009

Species cluster 11 (unnamed, 2 genomes)

Unclassified isolates NFACC39-1 and NFACC45.

Species cluster 11. Clusters identified

Kind	Species cluster	Subspecies cluster	Preferred name	Deposit	Authority
user strain	14	14	Pseudomonas sp NFACC39-1	-	-
user strain	14	14	Pseudomonas sp NFACC45	-	-
type strain	13	13	Pseudomonas chlororaphis subsp. piscium	DSM 21509	Burr et al. 2010
type strain	12	12	Pseudomonas lini	DSM 16768	Delorme et al. 2002
type strain	11	11	Pseudomonas taetrolens	DSM 21104	Haynes 1957
type strain	10	10	Pseudomonas corrugata	NCPPB 2445	Roberts and Scarlett 1981 emend. Sutra et al. 1997
type strain	10	10	Pseudomonas corrugata	DSM 7228	Roberts and Scarlett 1981 emend. Sutra et al. 1997
type strain	9	9	Pseudomonas chlororaphis	LMG 5004	(Guignard and Sauvageau 1894) Bergey et al. 1930 emend. Peix et al. 2007
type strain	8	8	Pseudomonas kilonensis	DSM 13647	Sikorski et al. 2001
type strain	7	7	Pseudomonas fluorescens	DSM 50090	Migula 1895
type strain	6	5	Pseudomonas mediterranea	CFBP 5447	Catara et al. 2002
type strain	5	4	Pseudomonas thivervalensis	LMG 21626	Achouak et al. 2000
type strain	4	3	Pseudomonas frederiksbergensis	LMG 19851	Andersen et al. 2000
type strain	3	2	Pseudomonas koreensis	LMG 21318	Kwon et al. 2003
type strain	2	1	Pseudomonas chlororaphis subsp. aurantiaca	DSM 19603	(Nakhimovskaya 1948) Peix et al. 2007
type strain	2	6	Pseudomonas chlororaphis subsp. aureofaciens	NBRC 3521	(Kluyver 1956) Peix et al. 2007
type strain	1	0	Pseudomonas brassicacearum	LMG 21623	Achouak et al. 2000 emend. Ivanova et al. 2009

Species cluster 10 (unnamed, 2 genomes)

Misclassified *P. frederiksbergensis* strain SI8 and misclassified *P. fluorescens* NT0133.

Species cluster 10. Clusters identified

Kind	Species cluster	Subspecies cluster	Preferred name	Deposit	Authority
user strain	16	16	Pseudomonas fluorescens NT0133	-	-
user strain	16	16	Pseudomonas frederiksbergensis SI8	-	-
type strain	15	15	Pseudomonas congelans	DSM 14939	Behrendt et al. 2003
type strain	14	14	Pseudomonas caricapapayae	ICMP 2855	Robbs 1956
type strain	13	13	Pseudomonas lini	DSM 16768	Delorme et al. 2002
type strain	12	11	Pseudomonas corrugata	NCPPB 2445	Roberts and Scarlett 1981 emend. Sutra et al. 1997
type strain	12	11	Pseudomonas corrugata	DSM 7228	Roberts and Scarlett 1981 emend. Sutra et al. 1997
type strain	11	10	Pseudomonas chlororaphis	LMG 5004	(Guignard and Sauvageau 1894) Bergey et al. 1930 emend. Peix et al. 2007
type strain	10	9	Pseudomonas kilonensis	DSM 13647	Sikorski et al. 2001
type strain	9	8	Pseudomonas mandelii	LMG 21607	Verhille et al. 1999
type strain	8	6	Pseudomonas mediterranea	CFBP 5447	Catara et al. 2002
type strain	7	5	Pseudomonas thivervalensis	LMG 21626	Achouak et al. 2000
type strain	6	4	Pseudomonas frederiksbergensis	LMG 19851	Andersen et al. 2000
type strain	5	3	Pseudomonas silesiensis	A3	Kaminski et al. 2018
type strain	4	12	Pseudomonas amygdali	CFBP 3205	Psallidas and Panagopoulos 1975
type strain	4	12	Pseudomonas meliae	CFBP 3225	Ogimi 1981
type strain	4	12	Pseudomonas savastanoi	ICMP 4352	(Janse 1982) Gardan et al. 1992
type strain	3	2	Pseudomonas chlororaphis subsp. aurantiaca	DSM 19603	(Nakhimovskaya 1948) Peix et al. 2007
type strain	3	7	Pseudomonas chlororaphis subsp. aureofaciens	NBRC 3521	(Kluyver 1956) Peix et al. 2007
type strain	2	1	Pseudomonas brassicacearum	LMG 21623	Achouak et al. 2000 emend. Ivanova et al. 2009
type strain	1	0	Pseudomonas caspiana	FBF102	Busquets et al. 2017

Species cluster 9 (unnamed, 1 genomes)

Misclassified *P. fluorescens* Pf29Arp.

Species cluster 9. Clusters identified

Kind	Species cluster	Subspecies cluster	Preferred name	Deposit	Authority
user strain	11	11	Pseudomonas fluorescens Pf29Arp	-	-
type strain	10	10	Pseudomonas lini	DSM 16768	Delorme et al. 2002
type strain	9	9	Pseudomonas corrugata	NCPPB 2445	Roberts and Scarlett 1981 emend. Sutra et al. 1997
type strain	9	9	Pseudomonas corrugata	DSM 7228	Roberts and Scarlett 1981 emend. Sutra et al. 1997
type strain	8	8	Pseudomonas chlororaphis	LMG 5004	(Guignard and Sauvageau 1894) Bergey et al. 1930 emend. Peix et al. 2007
type strain	7	7	Pseudomonas kilonensis	DSM 13647	Sikorski et al. 2001
type strain	6	5	Pseudomonas mediterranea	CFBP 5447	Catara et al. 2002
type strain	5	4	Pseudomonas thivervalensis	LMG 21626	Achouak et al. 2000
type strain	4	3	Pseudomonas migulae	NBRC 103157	Verhille et al. 1999
type strain	3	2	Pseudomonas frederiksbergensis	LMG 19851	Andersen et al. 2000
type strain	2	1	Pseudomonas chlororaphis subsp. aurantiaca	DSM 19603	(Nakhimovskaya 1948) Peix et al. 2007
type strain	2	6	Pseudomonas chlororaphis subsp. aureofaciens	NBRC 3521	(Kluyver 1956) Peix et al. 2007
type strain	1	0	Pseudomonas brassicacearum	LMG 21623	Achouak et al. 2000 emend. Ivanova et al. 2009

Species cluster 8 (unnamed, 4 genomes)

Misclassified *P. chlororaphis* strain UFB2, misclassified *P. brassicacearum* strain Wood3, misclassified *P. fluorescens* strain UM270 and unclassified isolate P97_38.

Species cluster 8. Clusters identified

Kind	Species cluster	Subspecies cluster	Preferred name	Deposit	Authority
user strain	20	20	Pseudomonas brassicacearum Wood3	-	-
user strain	20	20	Pseudomonas chlororaphis UFB2	-	-
user strain	20	20	Pseudomonas fluorescens UM270	-	-
user strain	20	20	Pseudomonas sp P97 38	-	-
type strain	19	19	Pseudomonas mucidolens	LMG 2223	Levine and Anderson 1932
type strain	18	18	Pseudomonas chlororaphis subsp. piscium	DSM 21509	Burr et al. 2010
type strain	17	17	Pseudomonas corrugata	NCPPB 2445	Roberts and Scarlett 1981 emend. Sutra et al. 1997
type strain	17	17	Pseudomonas corrugata	DSM 7228	Roberts and Scarlett 1981 emend. Sutra et al. 1997
type strain	16	16	Pseudomonas chlororaphis	LMG 5004	(Guignard and Sauvageau 1894) Bergey et al. 1930 emend. Peix et al. 2007
type strain	15	15	Pseudomonas azotoformans	LMG 21611	Iizuka and Komagata 1963
type strain	14	14	Pseudomonas kilonensis	DSM 13647	Sikorski et al. 2001
type strain	13	13	Pseudomonas libanensis	DSM 17149	Dabboussi et al. 1999
type strain	12	12	Pseudomonas synxantha	DSM 18928	(Ehrenberg 1840) Holland 1920
type strain	11	10	Pseudomonas protegens	CHA0	Ramette et al. 2012
type strain	10	9	Pseudomonas mediterranea	CFBP 5447	Catara et al. 2002
type strain	9	8	Pseudomonas thivervalensis	LMG 21626	Achouak et al. 2000
type strain	8	7	Pseudomonas lactis	DSM 29167	von Neubeck et al. 2017
type strain	7	6	Pseudomonas proteolytica	LMG 22710	Reddy et al. 2004 emend. van den Beld et al. 2016
type strain	6	5	Pseudomonas moraviensis	LMG 24280	Tvrzová et al. 2006
type strain	5	4	Pseudomonas gessardii	DSM 17152	Verhille et al. 1999 emend. van den Beld et al. 2016
type strain	4	3	Pseudomonas cedrina	DSM 17516	Dabboussi et al. 2002 emend. Behrendt et al. 2009
type strain	3	2	Pseudomonas kribbensis	KCTC 32541T	Chang et al. 2016
type strain	2	1	Pseudomonas chlororaphis subsp. aurantiaca	DSM 19603	(Nakhimovskaya 1948) Peix et al. 2007
type strain	2	11	Pseudomonas chlororaphis subsp. aureofaciens	NBRC 3521	(Kluyver 1956) Peix et al. 2007
type strain	1	0	Pseudomonas brassicacearum	LMG 21623	Achouak et al. 2000 emend. Ivanova et al. 2009

Species cluster 7 (unnamed, 8 genomes)

Unclassified isolates NFACC47-1, NFACC43, 7SR1, NFACC09-4, AF76, NFACC36, NFACC49-2 and NFACC32-1.

Species cluster 7. Clusters identified

Kind	Species cluster	Subspecies cluster	Preferred name	Deposit	Authority
user strain	20	20	Pseudomonas sp 7SR1	-	-
user strain	20	20	Pseudomonas sp AF76	-	-
user strain	20	20	Pseudomonas sp NFACC09-4	-	-
user strain	20	20	Pseudomonas sp NFACC32-1	-	-
user strain	20	20	Pseudomonas sp NFACC36	-	-
user strain	20	20	Pseudomonas sp NFACC43	-	-
user strain	20	20	Pseudomonas sp NFACC47-1	-	-
user strain	20	20	Pseudomonas sp NFACC49-2	-	-
type strain	19	19	Pseudomonas caricapapayae	ICMP 2855	Robbs 1956
type strain	18	18	Pseudomonas chlororaphis subsp. piscium	DSM 21509	Burr et al. 2010
type strain	17	17	Pseudomonas lini	DSM 16768	Delorme et al. 2002
type strain	16	16	Pseudomonas prosekii	LMG 26867	Kosina et al. 2014
type strain	15	13	Pseudomonas chlororaphis	LMG 5004	(Guignard and Sauvageau 1894) Bergey et al. 1930 emend. Peix et al. 2007
type strain	14	14	Pseudomonas corrugata	NCPPB 2445	Roberts and Scarlett 1981 emend. Sutra et al. 1997
type strain	14	14	Pseudomonas corrugata	DSM 7228	Roberts and Scarlett 1981 emend. Sutra et al. 1997
type strain	13	12	Pseudomonas kilonensis	DSM 13647	Sikorski et al. 2001
type strain	12	11	Pseudomonas veronii	DSM 11331	Elomari et al. 1996
type strain	11	10	Pseudomonas arsenicoxydans	CECT 7543	Campos et al. 2011
type strain	10	9	Pseudomonas mandelii	LMG 21607	Verhille et al. 1999
type strain	9	7	Pseudomonas mediterranea	CFBP 5447	Catara et al. 2002
type strain	8	6	Pseudomonas thivervalensis	LMG 21626	Achouak et al. 2000
type strain	7	5	Pseudomonas frederiksbergensis	LMG 19851	Andersen et al. 2000
type strain	6	4	Pseudomonas silesiensis	A3	Kaminski et al. 2018
type strain	5	3	Pseudomonas koreensis	LMG 21318	Kwon et al. 2003
type strain	4	15	Pseudomonas amygdali	CFBP 3205	Psallidas and Panagopoulos 1975
type strain	4	15	Pseudomonas meliae	CFBP 3225	Ogimi 1981
type strain	4	15	Pseudomonas savastanoi	ICMP 4352	(Janse 1982) Gardan et al. 1992
type strain	3	2	Pseudomonas laurylsulfatiphila	AP3_16	Furmanczyk et al. 2019
type strain	2	1	Pseudomonas chlororaphis subsp. aurantiaca	DSM 19603	(Nakhimovskaya 1948) Peix et al. 2007
type strain	2	8	Pseudomonas chlororaphis subsp. aureofaciens	NBRC 3521	(Kluyver 1956) Peix et al. 2007
type strain	1	0	Pseudomonas brassicacearum	LMG 21623	Achouak et al. 2000 emend. Ivanova et al. 2009

Species cluster 6 (unnamed, 1 genome)

Unclassified isolate Pf153.

Species cluster 6. Clusters identified

Kind	Species cluster	Subspecies cluster	Preferred name	Deposit	Authority
user strain	12	11	Pseudomonas sp Pf153	-	-
type strain	11	10	Pseudomonas chlororaphis subsp. piscium	DSM 21509	Burr et al. 2010
type strain	10	9	Pseudomonas lini	DSM 16768	Delorme et al. 2002
type strain	9	7	Pseudomonas chlororaphis	LMG 5004	(Guignard and Sauvageau 1894) Bergey et al. 1930 emend. Peix et al. 2007
type strain	8	8	Pseudomonas corrugata	NCPPB 2445	Roberts and Scarlett 1981 emend. Sutra et al. 1997
type strain	8	8	Pseudomonas corrugata	DSM 7228	Roberts and Scarlett 1981 emend. Sutra et al. 1997
type strain	7	6	Pseudomonas kilonensis	DSM 13647	Sikorski et al. 2001
type strain	6	5	Pseudomonas mediterranea	CFBP 5447	Catara et al. 2002
type strain	5	4	Pseudomonas thivervalensis	LMG 21626	Achouak et al. 2000
type strain	4	3	Pseudomonas rhodesiae	LMG 17764	Coroler et al. 1997
type strain	3	2	Pseudomonas chlororaphis subsp. aurantiaca	DSM 19603	(Nakhimovskaya 1948) Peix et al. 2007
type strain	2	1	Pseudomonas brassicacearum	LMG 21623	Achouak et al. 2000 emend. Ivanova et al. 2009
type strain	1	0	Pseudomonas grimontii	DSM 17515	Baïda et al. 2002

Species cluster 5 (*P. corrugata*, 7 genomes)

P. corrugata strains TEIC1148, CFBP5403, CFBP 5454, **NCPPB2445** [T], RM1-1-4, LMG 2172 and **DSM 7228** [T].

Species cluster 5 (*P. corrugata*). Clusters identified

Kind	Species cluster	Subspecies cluster	Preferred name	Deposit	Authority
type strain	9	12	Pseudomonas corrugata	NCPPB 2445	Roberts and Scarlett 1981 emend. Sutra et al. 1997
type strain	9	12	Pseudomonas corrugata	DSM 7228	Roberts and Scarlett 1981 emend. Sutra et al. 1997
user strain	9	12	Pseudomonas corrugata CFBP 5454	-	-
user strain	9	12	Pseudomonas corrugata CFBP5403	-	-
user strain	9	12	Pseudomonas corrugata DSM 7228 T	-	-
user strain	9	12	Pseudomonas corrugata LMG 2172	-	-
user strain	9	12	Pseudomonas corrugata NCPPB2445 T	-	-
user strain	9	12	Pseudomonas corrugata RM1-1-4	-	-
user strain	9	12	Pseudomonas corrugata TEIC1148	-	-
type strain	12	11	Pseudomonas chlororaphis subsp. piscium	DSM 21509	Burr et al. 2010
type strain	11	10	Pseudomonas lini	DSM 16768	Delorme et al. 2002
type strain	10	9	Pseudomonas taetrolens	DSM 21104	Haynes 1957
type strain	8	8	Pseudomonas chlororaphis	LMG 5004	(Guignard and Sauvageau 1894) Bergey et al. 1930 emend. Peix et al. 2007
type strain	7	7	Pseudomonas kilonensis	DSM 13647	Sikorski et al. 2001
type strain	6	6	Pseudomonas arsenicoxydans	CECT 7543	Campos et al. 2011
type strain	2	5	Pseudomonas chlororaphis subsp. aureofaciens	NBRC 3521	(Kluyver 1956) Peix et al. 2007
type strain	5	4	Pseudomonas mediterranea	CFBP 5447	Catara et al. 2002
type strain	4	3	Pseudomonas thivervalensis	LMG 21626	Achouak et al. 2000
type strain	3	2	Pseudomonas migulae	NBRC 103157	Verhille et al. 1999
type strain	2	1	Pseudomonas chlororaphis subsp. aurantiaca	DSM 19603	(Nakhimovskaya 1948) Peix et al. 2007
type strain	1	0	Pseudomonas brassicacearum	LMG 21623	Achouak et al. 2000 emend. Ivanova et al. 2009

Species cluster 4 (unnamed, 1 genome)

Unclassified isolate ICMP22404.

Species cluster 4. Clusters identified

Kind	Species cluster	Subspecies cluster	Preferred name	Deposit	Authority
user strain	11	11	Pseudomonas sp ICMP22404	-	-
type strain	10	10	Pseudomonas chlororaphis subsp. piscium	DSM 21509	Burr et al. 2010
type strain	9	9	Pseudomonas lini	DSM 16768	Delorme et al. 2002
type strain	8	8	Pseudomonas corrugata	NCPPB 2445	Roberts and Scarlett 1981 emend. Sutra et al. 1997
type strain	8	8	Pseudomonas corrugata	DSM 7228	Roberts and Scarlett 1981 emend. Sutra et al. 1997
type strain	7	7	Pseudomonas chlororaphis	LMG 5004	(Guignard and Sauvageau 1894) Bergey et al. 1930 emend. Peix et al. 2007
type strain	6	6	Pseudomonas kilonensis	DSM 13647	Sikorski et al. 2001
type strain	5	4	Pseudomonas mediterranea	CFBP 5447	Catara et al. 2002
type strain	4	3	Pseudomonas thivervalensis	LMG 21626	Achouak et al. 2000
type strain	3	2	Pseudomonas chlororaphis subsp. aurantiaca	DSM 19603	(Nakhimovskaya 1948) Peix et al. 2007
type strain	3	5	Pseudomonas chlororaphis subsp. aureofaciens	NBRC 3521	(Kluyver 1956) Peix et al. 2007
type strain	2	1	Pseudomonas brassicacearum	LMG 21623	Achouak et al. 2000 emend. Ivanova et al. 2009
type strain	1	0	Pseudomonas grimontii	DSM 17515	Baïda et al. 2002

Species cluster 3 (unnamed, 1 genome)

Unclassified isolate SHC52.

Species cluster 3. Clusters identified

Kind	Species cluster	Subspecies cluster	Preferred name	Deposit	Authority
user strain	10	9	Pseudomonas sp SHC52	-	-
type strain	9	8	Pseudomonas chlororaphis subsp. piscium	DSM 21509	Burr et al. 2010
type strain	8	7	Pseudomonas lini	DSM 16768	Delorme et al. 2002
type strain	7	6	Pseudomonas corrugata	NCPPB 2445	Roberts and Scarlett 1981 emend. Sutra et al. 1997
type strain	7	6	Pseudomonas corrugata	DSM 7228	Roberts and Scarlett 1981 emend. Sutra et al. 1997
type strain	6	5	Pseudomonas chlororaphis	LMG 5004	(Guignard and Sauvageau 1894) Bergey et al. 1930 emend. Peix et al. 2007
type strain	5	4	Pseudomonas kilonensis	DSM 13647	Sikorski et al. 2001
type strain	4	3	Pseudomonas mediterranea	CFBP 5447	Catara et al. 2002
type strain	3	2	Pseudomonas thivervalensis	LMG 21626	Achouak et al. 2000
type strain	2	1	Pseudomonas chlororaphis subsp. aurantiaca	DSM 19603	(Nakhimovskaya 1948) Peix et al. 2007
type strain	1	0	Pseudomonas brassicacearum	LMG 21623	Achouak et al. 2000 emend. Ivanova et al. 2009

Species cluster 2 (unnamed, 6 genomes)

Unclassified isolates SJZ085, SJZ074, SJZ078, SJZ075, SJZ124 and SJZ101.

Species cluster 2. Clusters identified

Kind	Species cluster	Subspecies cluster	Preferred name	Deposit	Authority
user strain	12	12	Pseudomonas sp SJZ074	-	-
user strain	12	12	Pseudomonas sp SJZ075	-	-
user strain	12	12	Pseudomonas sp SJZ078	-	-
user strain	12	12	Pseudomonas sp SJZ085	-	-
user strain	12	12	Pseudomonas sp SJZ101	-	-
user strain	12	12	Pseudomonas sp SJZ124	-	-
type strain	11	11	Pseudomonas chlororaphis subsp. piscium	DSM 21509	Burr et al. 2010
type strain	10	10	Pseudomonas lini	DSM 16768	Delorme et al. 2002
type strain	9	9	Pseudomonas prosekii	LMG 26867	Kosina et al. 2014
type strain	8	8	Pseudomonas corrugata	NCPPB 2445	Roberts and Scarlett 1981 emend. Sutra et al. 1997
type strain	8	8	Pseudomonas corrugata	DSM 7228	Roberts and Scarlett 1981 emend. Sutra et al. 1997
type strain	7	7	Pseudomonas chlororaphis	LMG 5004	(Guignard and Sauvageau 1894) Bergey et al. 1930 emend. Peix et al. 2007
type strain	6	6	Pseudomonas kilonensis	DSM 13647	Sikorski et al. 2001
type strain	5	4	Pseudomonas mediterranea	CFBP 5447	Catara et al. 2002
type strain	4	3	Pseudomonas thivervalensis	LMG 21626	Achouak et al. 2000
type strain	3	2	Pseudomonas chlororaphis subsp. aurantiaca	DSM 19603	(Nakhimovskaya 1948) Peix et al. 2007
type strain	3	5	Pseudomonas chlororaphis subsp. aureofaciens	NBRC 3521	(Kluyver 1956) Peix et al. 2007
type strain	2	1	Pseudomonas brassicacearum	LMG 21623	Achouak et al. 2000 emend. Ivanova et al. 2009
type strain	1	0	Pseudomonas grimontii	DSM 17515	Baïda et al. 2002

Species cluster 1 (*P. mediterranea*, 7 genomes)

P. mediterranea strains **DSM 16733** ^{T}, **CFBP 5447** ^{T}, TEIC1022, CFBP5404, CFBP5444 and TEIC1105 and unclassified isolate S58.

Species cluster 1 (*P. mediterranea*). Clusters identified

Kind	Species cluster	Subspecies cluster	Preferred name	Deposit	Authority
type strain	6	12	Pseudomonas mediterranea	CFBP 5447	Catara et al. 2002
user strain	6	12	Pseudomonas mediterranea CFBP 5447 T	-	-
user strain	6	12	Pseudomonas mediterranea CFBP5404	-	-
user strain	6	12	Pseudomonas mediterranea CFBP5444	-	-
user strain	6	12	Pseudomonas mediterranea DSM 16733 T	-	-
user strain	6	12	Pseudomonas mediterranea TEIC1022	-	-
user strain	6	12	Pseudomonas mediterranea TEIC1105	-	-
user strain	6	12	Pseudomonas sp S58	-	-
type strain	12	11	Pseudomonas chlororaphis subsp. piscium	DSM 21509	Burr et al. 2010
type strain	11	10	Pseudomonas lini	DSM 16768	Delorme et al. 2002
type strain	10	9	Pseudomonas prosekii	LMG 26867	Kosina et al. 2014
type strain	8	8	Pseudomonas corrugata	NCPPB 2445	Roberts and Scarlett 1981 emend. Sutra et al. 1997
type strain	8	8	Pseudomonas corrugata	DSM 7228	Roberts and Scarlett 1981 emend. Sutra et al. 1997
type strain	9	7	Pseudomonas chlororaphis	LMG 5004	(Guignard and Sauvageau 1894) Bergey et al. 1930 emend. Peix et al. 2007
type strain	7	6	Pseudomonas kilonensis	DSM 13647	Sikorski et al. 2001
type strain	3	5	Pseudomonas chlororaphis subsp. aureofaciens	NBRC 3521	(Kluyver 1956) Peix et al. 2007
type strain	5	4	Pseudomonas thivervalensis	LMG 21626	Achouak et al. 2000
type strain	4	3	Pseudomonas rhodesiae	LMG 17764	Coroler et al. 1997
type strain	3	2	Pseudomonas chlororaphis subsp. aurantiaca	DSM 19603	(Nakhimovskaya 1948) Peix et al. 2007
type strain	2	1	Pseudomonas brassicacearum	LMG 21623	Achouak et al. 2000 emend. Ivanova et al. 2009
type strain	1	0	Pseudomonas grimontii	DSM 17515	Baïda et al. 2002

References

Achouak, W., Sutra, L., Heulin, T., Meyer, J.-M., Fromin, N., Degraeve, S., et al. (2000). *Pseudomonas brassicacearum* sp. nov. and *Pseudomonas thivervalensis* sp. nov., two root-associated bacteria isolated from *Brassica napus* and *Arabidopsis thaliana*. *International Journal of Systematic and Evolutionary Microbiology* 50(1)**,** 9-18.

Andersen, S.M., Johnsen, K., Sørensen, J., Nielsen, P., and Jacobsen, C.S. (2000). *Pseudomonas frederiksbergensis* sp. nov., isolated from soil at a coal gasification site. *International Journal of Systematic and Evolutionary Microbiology* 50(6)**,** 1957-1964.

Arnau, V.G., Sánchez, L.A., and Delgado, O.D. (2015). *Pseudomonas yamanorum* sp. nov., a psychrotolerant bacterium isolated from a subantarctic environment. *International journal of systematic and evolutionary microbiology* 65(2)**,** 424-431.

Baïda, N., Yazourh, A., Singer, E., and Izard, D. (2002). *Pseudomonas grimontii* sp. nov. *International journal of systematic and evolutionary microbiology* 52(5)**,** 1497-1503.

Behrendt, U., Schumann, P., Meyer, J.-M., and Ulrich, A. (2009). *Pseudomonas cedrina* subsp. *fulgida* subsp. nov., a fluorescent bacterium isolated from the phyllosphere of grasses; emended description of *Pseudomonas cedrina* and description of *Pseudomonas cedrina* subsp. *cedrina* subsp. nov. *International journal of systematic and evolutionary microbiology* 59(6)**,** 1331-1335.

Behrendt, U., Ulrich, A., and Schumann, P. (2003). Fluorescent pseudomonads associated with the phyllosphere of grasses; *Pseudomonas trivialis* sp. nov., *Pseudomonas poae* sp. nov. and *Pseudomonas congelans* sp. nov. *Journal of Medical Microbiology* 53(5)**,** 1461-1469.

Burr, S.E., Gobeli, S., Kuhnert, P., Goldschmidt-Clermont, E., and Frey, J. (2010). *Pseudomonas chlororaphis* subsp. *piscium* subsp. nov., isolated from freshwater fish. *International journal of systematic and evolutionary microbiology* 60(12)**,** 2753-2757.

Busquets, A., Gomila, M., Beiki, F., Mulet, M., Rahimian, H., García-Valdés, E., et al. (2017). *Pseudomonas caspiana* sp. nov., a citrus pathogen in the *Pseudomonas syringae* phylogenetic group. *Systematic and Applied Microbiology* 40(5)**,** 266-273.

Campos, V.L., Valenzuela, C., Yarza, P., Kämpfer, P., Vidal, R., Zaror, C., et al. (2010). *Pseudomonas arsenicoxydans* sp nov., an arsenite-oxidizing strain isolated from the Atacama desert. *Systematic and Applied Microbiology* 33(4)**,** 193-197.

Catara, V., Sutra, L., Morineau, A., Achouak, W., Christen, R., and Gardan, L. (2002). Phenotypic and genomic evidence for the revision of *Pseudomonas corrugata* and proposal of *Pseudomonas mediterranea* sp. nov. *International Journal of Systematic and Evolutionary Microbiology* 52(5)**,** 1749-1758.

Coroler, L., Elomari, M., Hoste, B., Gillis, M., Izard, D., and Leclerc, H. (1996). *Pseudomonas rhodesiae* sp. nov., a new species isolated from natural mineral waters. *Systematic and applied Microbiology* 19(4)**,** 600-607.

Chang, D.-H., Rhee, M.-S., Kim, J.-S., Lee, Y., Park, M.Y., Kim, H., et al. (2016). *Pseudomonas kribbensis* sp. nov., isolated from garden soils in Daejeon, Korea. *Antonie Van Leeuwenhoek* 109(11)**,** 1433-1446.

Dabboussi, F., Hamze, M., Elomari, M., Verhille, S., Baida, N., Izard, D., et al. (1999a). *Pseudomonas libanensis* sp. nov., a new specie isolated from Lebanese spring waters. *International Journal of Systematic and Evolutionary Microbiology* 49(3)**,** 1091-1101.

Dabboussi, F., Hamze, M., Elomari, M., Verhille, S., Baida, N., Izard, D., et al. (1999b). Taxonomic study of bacteria isolated from Lebanese spring waters: proposal for *Pseudomonas cedrella* sp. nov. and *P. orientalis* sp. nov. *Research in microbiology* 150(5)**,** 303-316.

Delorme, S., Lemanceau, P., Christen, R., Corberand, T., Meyer, J.-M., and Gardan, L. (2002). *Pseudomonas lini* sp. nov., a novel species from bulk and rhizospheric soils. *International journal of systematic and evolutionary microbiology* 52(2)**,** 513-523.

Ehrenberg, C. (1840). Characteristik von 274 neuen Arten von Infusorien. *Bericht über die zur Bekanntmachung geeigneten Verhandlungen der Königlich Preussischen Akademie der Wissenschaften zu Berlin* 1840**,** 197-190.

Elomari, M., Coroler, L., Hoste, B., Gillis, M., Izard, D., and Leclerc, H. (1996). DNA relatedness among *Pseudomonas* strains isolated from natural mineral waters and proposal of *Pseudomonas veronii* sp. nov. *International Journal of Systematic and Evolutionary Microbiology* 46(4)**,** 1138-1144.

Furmanczyk, E.M., Kaminski, M.A., Lipinski, L., Dziembowski, A., and Sobczak, A. (2018). *Pseudomonas laurylsulfatovorans* sp. nov., sodium dodecyl sulfate degrading bacteria, isolated from the peaty soil of a wastewater treatment plant. *Systematic and applied microbiology* 41(4)**,** 348-354.

Gardan, L., Bella, P., Meyer, J.-M., Christen, R., Rott, P., Achouak, W., et al. (2002). *Pseudomonas salomonii* sp. nov., pathogenic on garlic, and *Pseudomonas palleroniana* sp. nov., isolated from rice. *International Journal of Systematic and Evolutionary Microbiology* 52(6)**,** 2065-2074.

Gardan, L., Bollet, C., Ghorrah, M.A., Grimont, F., and Grimont, P. (1992). DNA relatedness among the pathovar strains of *Pseudomonas syringae* subsp. *savastanoi* Janse (1982) and proposal of *Pseudomonas savastanoi* sp. nov. *International Journal of Systematic and Evolutionary Microbiology* 42(4)**,** 606-612.

Haynes, W., and Burkholder, W. (1957). Genus I. *Pseudomonas* Migula 1894. *Bergey's manual of determinative bacteriology***,** 89-152.

Hofmann, K., Huptas, C., Doll, E.V., Scherer, S., and Wenning, M. (2020). *Pseudomonas haemolytica* sp. nov., isolated from raw milk and skimmed milk concentrate. *International Journal of Systematic and Evolutionary Microbiology* 70(4)**,** 2339-2347.

Iizuka, h., and komagata, k. (1963). An attempt at grouping of the genus *Pseudomonas*. *The Journal of General and Applied Microbiology* 9(1)**,** 73-82.

Ivanova, E.P., Christen, R., Bizet, C., Clermont, D., Motreff, L., Bouchier, C., et al. (2009). *Pseudomonas brassicacearum* subsp. *neoaurantiaca* subsp. nov., orange-pigmented bacteria isolated from soil and the rhizosphere of agricultural plants. *International journal of systematic and evolutionary microbiology* 59(10)**,** 2476-2481.

Janse, J. (1982). *Pseudomonas syringae* subsp. *savastanoi* (ex Smith) subsp. nov., nom. rev., the bacterium causing excrescences on *Oleaceae* and *Nerium oleander* L. *International Journal of Systematic and Evolutionary Microbiology* 32(2)**,** 166-169.

Kaminski, M.A., Furmanczyk, E.M., Sobczak, A., Dziembowski, A., and Lipinski, L. (2018). *Pseudomonas silesiensis* sp. nov. strain A3T isolated from a biological pesticide sewage treatment plant and analysis of the complete genome sequence. *Systematic and applied microbiology* 41(1)**,** 13-22.

Kluyver, A. (1956). *Pseudomonas aureofaciens* nov. spec. and its pigments. *Journal of Bacteriology* 72(3)**,** 406.

Kosina, M., Barták, M., Mašlaňová, I., Pascutti, A.V., Šedo, O., Lexa, M., et al. (2013). *Pseudomonas prosekii* sp. nov., a novel psychrotrophic bacterium from Antarctica. *Current microbiology* 67(6)**,** 637-646.

Kreft, Ł., Botzki, A., Coppens, F., Vandepoele, K., and Van Bel, M. (2017). PhyD3: a phylogenetic tree viewer with extended phyloXML support for functional genomics data visualization. *Bioinformatics* 33(18)**,** 2946-2947.

Kwon, S.W., Kim, J.S., Park, I.C., Yoon, S.H., Park, D.H., Lim, C.K., et al. (2003). *Pseudomonas koreensis* sp. nov., *Pseudomonas umsongensis* sp. nov. and *Pseudomonas jinjuensis* sp. nov., novel species from farm soils in Korea. *International journal of systematic and evolutionary microbiology* 53(1)**,** 21-27.

Lagesen, K., Hallin, P., Rødland, E.A., Stærfeldt, H.-H., Rognes, T., and Ussery, D.W. (2007). RNAmmer: consistent and rapid annotation of ribosomal RNA genes. *Nucleic acids research* 35(9)**,** 3100-3108.

Lefort, V., Desper, R., and Gascuel, O. (2015). FastME 2.0: a comprehensive, accurate, and fast distance-based phylogeny inference program. *Molecular biology and evolution* 32(10)**,** 2798-2800.
Levine, M., and Anderson, D. (1932). Two new species of bacteria causing mustiness in eggs. *Journal of bacteriology* 23(4)**,** 337.
Liu, Y., Lai, Q., Göker, M., Meier-Kolthoff, J.P., Wang, M., Sun, Y., et al. (2015). Genomic insights into the taxonomic status of the *Bacillus cereus* group. *Scientific reports* 5(1)**,** 1-11.
López, N.I., Pettinari, M.J., Stackebrandt, E., Tribelli, P.M., Põtter, M., Steinbüchel, A., et al. (2009). *Pseudomonas extremaustralis* sp. nov., a poly (3-hydroxybutyrate) producer isolated from an Antarctic environment. *Current microbiology* 59(5)**,** 514-519.
Meier-Kolthoff, J.P., Auch, A.F., Klenk, H.-P., and Göker, M. (2013). Genome sequence-based species delimitation with confidence intervals and improved distance functions. *BMC bioinformatics* 14(1)**,** 60.
Meier-Kolthoff, J.P., and Göker, M. (2019). TYGS is an automated high-throughput platform for state-of-the-art genome-based taxonomy. *Nature communications* 10(1)**,** 1-10.
Migula, W. (1887). *Bacteriaceae (stäbchenbacterien).* W. Engelmann.
Morimoto, Y., Uwabe, K., Tohya, M., Hiramatsu, K., Kirikae, T., and Baba, T. (2020). *Pseudomonas atagosis* sp. nov., and *Pseudomonas akappagea* sp. nov., New Soil Bacteria Isolated from Samples on the Volcanic Island Izu Oshima, Tokyo. *Current Microbiology***,** 1-7.
Nakhimovskaya, M. (1948). *Pseudomonas aurantiaca* n. sp. *Mikrobiologiya* 17**,** 58-65.
Ogimi, C. (1977). Studies on bacterial gall of Chinaberry (*Melia Azedarach* Lin.), caused by *Pseudomonas meliae* n. sp. *Science Bulletin of the College of Agriculture-University of the Ryukyu (Japan)*.
Ondov, B.D., Treangen, T.J., Melsted, P., Mallonee, A.B., Bergman, N.H., Koren, S., et al. (2016). Mash: fast genome and metagenome distance estimation using MinHash. *Genome biology* 17(1)**,** 132.
Oueslati, M., Mulet, M., Gomila, M., Berge, O., Hajlaoui, M.R., Lalucat, J., et al. (2019). New species of pathogenic *Pseudomonas* isolated from citrus in Tunisia: Proposal of *Pseudomonas kairouanensis* sp. nov. and *Pseudomonas nabeulensis* sp. nov. *Systematic and applied microbiology* 42(3)**,** 348-359.
Paine, S.G. (1919). Studies in bacteriosis. II. A brown blotch disease of cultivated mushrooms. *Annals of Applied Biology* 5**,** 206-219.
Pascual, J., García-López, M., Bills, G.F., and Genilloud, O. (2015). *Pseudomonas granadensis* sp. nov., a new bacterial species isolated from the Tejeda, Almijara and Alhama Natural Park, Granada, Spain. *International journal of systematic and evolutionary microbiology* 65(2)**,** 625-632.
Peix, A., Valverde, A., Rivas, R., Igual, J.M., Ramírez-Bahena, M.-H., Mateos, P.F., et al. (2007). Reclassification of *Pseudomonas aurantiaca* as a synonym of *Pseudomonas chlororaphis* and proposal of three subspecies, *P. chlororaphis* subsp. *chlororaphis* subsp. nov., *P. chlororaphis* subsp. *aureofaciens* subsp. nov., comb. nov. and *P. chlororaphis* subsp. *aurantiaca* subsp. nov., comb. nov. *International journal of systematic and evolutionary microbiology* 57(6)**,** 1286-1290.
Psallidas, P., and Panagopoulos, C. (1975). A new bacteriosis of almond caused by *Pseudomonas amygdali* sp. nov.
Ramette, A., Frapolli, M., Fischer-Le Saux, M., Gruffaz, C., Meyer, J.-M., Défago, G., et al. (2011). *Pseudomonas protegens* sp. nov., widespread plant-protecting bacteria producing the biocontrol compounds 2, 4-diacetylphloroglucinol and pyoluteorin. *Systematic and applied microbiology* 34(3)**,** 180-188.
Reddy, G.S., Matsumoto, G.I., Schumann, P., Stackebrandt, E., and Shivaji, S. (2004). Psychrophilic pseudomonads from Antarctica: *Pseudomonas antarctica* sp. nov., *Pseudomonas meridiana* sp. nov. and *Pseudomonas proteolytica* sp. nov. *International journal of systematic and evolutionary microbiology* 54(3)**,** 713-719.
Robbs, C. (1956). Uma nova doença bacteriana do mamoeiro (*Carica papaya* L.). *Revist. Soc. Brasil. Agron* 12**,** 73-76.

Roberts, P., and Scarlett, C. (1981). *Pseudomonas corrugata* sp. nov. I Validation of the publication of new names and new combinations previously effectively published outside the IJSB. List No. 6. *Int. J. Syst. Bacteriol.* 31**,** 216.

Sikorski, J., Stackebrandt, E., and Wackernagel, W. (2001). *Pseudomonas kilonensis* sp. nov., a bacterium isolated from agricultural soil. *International Journal of Systematic and Evolutionary Microbiology* 51(4)**,** 1549-1555.

Sutra, L., Siverio, F., Lopez, M., Hunault, G., Bollet, C., and Gardan, L. (1997). Taxonomy of *Pseudomonas* strains isolated from tomato pith necrosis: emended description of *Pseudomonas corrugata* and proposal of three unnamed fluorescent *Pseudomonas* genomospecies. *International Journal of Systematic and Evolutionary Microbiology* 47(4)**,** 1020-1033.

Tvrzova, L., Schumann, P., Spröer, C., Sedláček, I., Páčová, Z., Šedo, O., et al. (2006). *Pseudomonas moraviensis* sp. nov. and *Pseudomonas vranovensis* sp. nov., soil bacteria isolated on nitroaromatic compounds, and emended description of *Pseudomonas asplenii*. *International Journal of Systematic and Evolutionary Microbiology* 56(11)**,** 2657-2663.

van den Beld, M.J., Reinders, E., Notermans, D.W., and Reubsaet, F.A. (2016). Possible misidentification of species in the *Pseudomonas fluorescens* lineage as *Burkholderia pseudomallei* and *Francisella tularensis*, and emended descriptions of *Pseudomonas brenneri*, *Pseudomonas gessardii* and *Pseudomonas proteolytica*. *International Journal of Systematic and Evolutionary Microbiology* 66(9)**,** 3420-3425.

Verhille, S., Baida, N., Dabboussi, F., Hamze, M., Izard, D., and Leclerc, H. (1999). *Pseudomonas gessardii* sp. nov. and *Pseudomonas migulae* sp. nov., two new species isolated from natural mineral waters. *International Journal of Systematic and Evolutionary Microbiology* 49(4)**,** 1559-1572.

von Neubeck, M., Huptas, C., Glück, C., Krewinkel, M., Stoeckel, M., Stressler, T., et al. (2017). *Pseudomonas lactis* sp. nov. and *Pseudomonas paralactis* sp. nov., isolated from bovine raw milk. *International journal of systematic and evolutionary microbiology* 67(6)**,** 1656-1664.

Supplementary File S6. Separation of PCA observational groups and significance based on the first two principal components' centroids.

Group 1	Group 2	Mahalanobis distance	Two-sample T^2 statistic	F-value	Critical F-value	Significant?[a]
1	2	1.90337	4.34737	1.44912	19	No
1	5	0.67481	0.68305	0.25614	9.55209	No
1	6	13.99652	146.927	36.73175	199.5	No
1	7	41.50021	3757.674	1670.077	4.45897	Yes
1	8	14.21648	242.5298	80.84327	19	Yes
1	9	19.12462	274.3135	68.57836	199.5	No
1	10	7.39275	40.98956	10.24739	199.5	No
1	11	12.80986	196.911	65.637	19	Yes
1	12	13.87159	329.8647	131.9459	6.94427	Yes
1	13	43.47548	2268.141	756.047	19	Yes
1	14	26.33439	520.1252	130.0313	199.5	No
1	15	20.44033	313.3552	78.33879	199.5	No
1	16	57.51588	2481.057	620.2642	199.5	Yes
1	17	38.32018	1101.327	275.3317	199.5	Yes
1	18	63.39555	4822.795	1607.598	19	Yes
1	19	41.4096	2572.133	964.5498	9.55209	Yes
1	20	20.36466	311.0394	77.75985	199.5	No
1	22	58.50592	2567.207	641.8018	199.5	Yes
1	23	37.23368	2599.4	1083.083	5.78614	Yes
1	24	64.59018	9102.308	4045.47	4.45897	Yes
1	25	15.18974	346.0922	129.7846	9.55209	Yes
1	26	58.50351	5133.99	1925.246	9.55209	Yes
1	27	33.08096	1641.525	615.5717	9.55209	Yes
1	28	15.51157	180.4566	45.11415	199.5	No
1	29	43.46419	4667.276	2178.062	3.73889	Yes
2	5	0.62998	0.47625	0.15875	19	No
2	6	5.86839	22.95867	0	NA	NA
2	7	27.54423	1213.895	531.0793	4.73741	Yes
2	8	13.07948	171.0729	42.76822	199.5	No
2	9	24.75077	408.4006	0	NA	NA
2	10	13.80352	127.0247	0	NA	NA
2	11	8.00788	64.12619	16.03155	199.5	No
2	12	11.00621	161.5156	60.56836	9.55209	Yes
2	13	52.30154	2735.451	683.8628	199.5	Yes
2	14	38.09833	967.6554	0	NA	NA
2	15	25.82908	444.7611	0	NA	NA
2	16	70.10589	3276.557	0	NA	NA
2	17	41.80681	1165.206	0	NA	NA
2	18	75.03011	5629.518	1407.38	199.5	Yes
2	19	51.46493	3178.367	1059.456	19	Yes
2	20	22.42764	335.3327	0	NA	NA
2	22	75.27317	3777.367	0	NA	NA
2	23	44.53213	2833.015	1133.206	6.94427	Yes
2	24	71.72886	8232.046	3601.52	4.73741	Yes
2	25	23.2316	647.6486	215.8829	19	Yes
2	26	74.3403	6631.776	2210.592	19	Yes
2	27	34.03965	1390.437	463.479	19	Yes
2	28	23.95785	382.6524	0	NA	NA

2	29	59.48163	6191.613	2874.677	3.80557	Yes
5	6	8.79628	58.03086	14.50771	199.5	No
5	7	31.61552	2180.816	969.2518	4.45897	Yes
5	8	10.30732	127.4891	42.49638	19	Yes
5	9	18.21151	248.7442	62.18606	199.5	No
5	10	8.54915	54.81601	13.704	199.5	No
5	11	7.84654	73.88186	24.62729	19	Yes
5	12	9.33543	149.4004	59.76016	6.94427	Yes
5	13	42.57279	2174.931	724.977	19	Yes
5	14	28.96299	629.1409	157.2852	199.5	No
5	15	19.25355	278.0243	69.50608	199.5	No
5	16	58.18446	2539.074	634.7685	199.5	Yes
5	17	34.62942	899.3974	224.8493	199.5	Yes
5	18	63.05615	4771.294	1590.431	19	Yes
5	19	41.45586	2577.882	966.7058	9.55209	Yes
5	20	17.33198	225.2982	56.32455	199.5	No
5	22	62.2278	2904.224	726.0561	199.5	Yes
5	23	35.81575	2405.189	1002.162	5.78614	Yes
5	24	61.38629	8221.695	3654.087	4.45897	Yes
5	25	16.31887	399.4584	149.7969	9.55209	Yes
5	26	61.48492	5670.594	2126.473	9.55209	Yes
5	27	28.45105	1214.193	455.3225	9.55209	Yes
5	28	16.89424	214.0614	53.51536	199.5	No
5	29	47.86961	5661.352	2641.964	3.73889	Yes
6	7	8.56853	65.26194	27.9694	5.14325	Yes
6	8	13.83577	127.619	0	NA	NA
6	9	35.58825	633.2617	-Inf	NA	NA
6	10	29.43034	433.0723	-Inf	NA	NA
6	11	4.49809	13.48852	0	NA	NA
6	12	9.47762	71.86025	23.95342	19	Yes
6	13	64.95085	2812.409	0	NA	NA
6	14	59.81429	1788.875	-Inf	NA	NA
6	15	35.95849	646.5067	-Inf	NA	NA
6	16	88.16369	3886.418	-Inf	NA	NA
6	17	45.60848	1040.067	-Inf	NA	NA
6	18	90.43453	5452.27	0	NA	NA
6	19	66.79667	3346.347	836.5867	199.5	Yes
6	20	26.81637	359.5589	-Inf	NA	NA
6	22	102.0861	5210.782	-Inf	NA	NA
6	23	55.24383	2543.234	953.7126	9.55209	Yes
6	24	78.59683	5491.077	2353.319	5.14325	Yes
6	25	39.71347	1182.87	295.7174	199.5	Yes
6	26	99.07616	7362.064	1840.516	199.5	Yes
6	27	34.45304	890.2589	222.5647	199.5	Yes
6	28	41.1989	848.6747	-Inf	NA	NA
6	29	87.31203	7115.164	3283.922	3.88529	Yes
7	8	22.0688	779.2512	340.9224	4.73741	Yes
7	9	51.92972	2397.063	1027.313	5.14325	Yes
7	10	55.02432	2691.268	1153.4	5.14325	Yes
7	11	11.0856	196.625	86.02343	4.73741	Yes
7	12	16.30773	709.1792	319.1307	4.25649	Yes
7	13	78.33659	9818.594	4295.635	4.73741	Yes

7	14	87.48567	6803.326	2915.711	5.14325	Yes
7	15	51.20202	2330.352	998.7225	5.14325	Yes
7	16	105.5981	9911.956	4247.981	5.14325	Yes
7	17	50.55215	2271.573	973.5313	5.14325	Yes
7	18	103.9009	17272.64	7556.782	4.73741	Yes
7	19	83.60818	15251.62	6778.499	4.45897	Yes
7	20	36.27403	1169.605	501.2592	5.14325	Yes
7	22	130.3345	15099.63	6471.271	5.14325	Yes
7	23	67.57754	14051.46	6387.027	4.10282	Yes
7	24	82.45984	27198.5	12627.88	3.80557	Yes
7	25	63.64355	8837.457	3927.758	4.45897	Yes
7	26	124.6245	33886.39	15060.62	4.45897	Yes
7	27	37.23824	3025.497	1344.666	4.45897	Yes
7	28	65.97286	3868.816	1658.064	5.14325	Yes
7	29	119.5644	72777.78	34569.45	3.52189	Yes
8	9	6.31466	26.58331	0	NA	NA
8	10	9.53423	60.60102	0	NA	NA
8	11	2.72126	7.40528	1.85132	199.5	No
8	12	0.48643	0.31548	0.11831	9.55209	No
8	13	19.2465	370.4277	92.60692	199.5	No
8	14	21.8664	318.7596	0	NA	NA
8	15	6.13392	25.0833	0	NA	NA
8	16	33.24408	736.7792	0	NA	NA
8	17	9.35026	58.28493	0	NA	NA
8	18	33.90335	1149.437	287.3594	199.5	Yes
8	19	20.9092	524.6336	174.8779	19	Yes
8	20	2.16666	3.12962	0	NA	NA
8	22	45.43268	1376.086	0	NA	NA
8	23	14.0738	282.9598	113.1839	6.94427	Yes
8	24	26.66694	1137.801	497.788	4.73741	Yes
8	25	11.23076	151.356	50.452	19	Yes
8	26	42.41749	2159.092	719.6975	19	Yes
8	27	5.13735	31.67078	10.55693	19	No
8	28	12.27546	100.4579	0	NA	NA
8	29	38.95803	2656.024	1233.154	3.80557	Yes
9	10	4.02629	8.10552	-Inf	NA	NA
9	11	16.63109	184.3953	0	NA	NA
9	12	10.21158	83.42103	27.80701	19	Yes
9	13	5.09987	17.33911	0	NA	NA
9	14	5.17564	13.39363	-Inf	NA	NA
9	15	0.04024	0.00081	-Inf	NA	NA
9	16	11.9419	71.30452	-Inf	NA	NA
9	17	4.6166	10.65649	-Inf	NA	NA
9	18	13.61388	123.5585	0	NA	NA
9	19	5.00062	18.75462	4.68866	199.5	No
9	20	1.79185	1.60536	-Inf	NA	NA
9	22	17.917	160.5095	-Inf	NA	NA
9	23	2.99285	7.46429	2.79911	9.55209	No
9	24	13.62137	164.9261	70.6826	5.14325	Yes
9	25	1.68765	2.13611	0.53403	199.5	No
9	26	16.2062	196.9808	49.2452	199.5	No
9	27	5.36275	21.56928	5.39232	199.5	No

9	28	2.07527	2.15338	-Inf	NA	NA
9	29	14.18262	187.7371	86.64787	3.88529	Yes
10	11	16.71976	186.367	0	NA	NA
10	12	12.56904	126.3846	42.12821	19	Yes
10	13	16.16465	174.1973	0	NA	NA
10	14	6.042	18.25291	-Inf	NA	NA
10	15	4.83534	11.69027	-Inf	NA	NA
10	16	23.91044	285.8546	-Inf	NA	NA
10	17	17.04436	145.2551	-Inf	NA	NA
10	18	28.23467	531.4646	0	NA	NA
10	19	14.29895	153.345	38.33624	199.5	No
10	20	7.95294	31.62465	-Inf	NA	NA
10	22	24.69359	304.8866	-Inf	NA	NA
10	23	13.11149	143.2593	53.72225	9.55209	Yes
10	24	31.83511	900.866	386.0854	5.14325	Yes
10	25	1.40323	1.4768	0.3692	199.5	No
10	26	24.42481	447.4284	111.8571	199.5	No
10	27	16.63402	207.518	51.87949	199.5	No
10	28	1.48724	1.10594	-Inf	NA	NA
10	29	16.06466	240.8684	111.17	3.88529	Yes
11	12	0.94362	1.18724	0.44521	9.55209	No
11	13	36.435	1327.509	331.8773	199.5	Yes
11	14	37.15884	920.5195	0	NA	NA
11	15	16.57634	183.1833	0	NA	NA
11	16	54.88357	2008.137	0	NA	NA
11	17	21.52742	308.9531	0	NA	NA
11	18	55.83223	3117.238	779.3094	199.5	Yes
11	19	38.53599	1782.027	594.009	19	Yes
11	20	9.7423	63.27495	0	NA	NA
11	22	68.96083	3170.397	0	NA	NA
11	23	29.17052	1215.599	486.2397	6.94427	Yes
11	24	45.58049	3324.13	1454.307	4.73741	Yes
11	25	21.99512	580.5424	193.5141	19	Yes
11	26	65.66558	5174.362	1724.787	19	Yes
11	27	14.0545	237.0346	79.01155	19	Yes
11	28	23.32069	362.5699	0	NA	NA
11	29	59.27759	6149.207	2854.989	3.80557	Yes
12	13	25.78485	886.4782	332.4293	9.55209	Yes
12	14	28.25751	638.7897	212.9299	19	Yes
12	15	10.0342	80.54809	26.84936	19	Yes
12	16	41.75072	1394.498	464.8327	19	Yes
12	17	13.50486	145.9049	48.63498	19	Yes
12	18	42.35529	2391.961	896.9852	9.55209	Yes
12	19	27.77206	1322.207	528.8829	6.94427	Yes
12	20	4.662	17.38738	5.79579	19	No
12	22	55.16723	2434.739	811.5795	19	Yes
12	23	19.72387	864.5135	370.5058	5.14325	Yes
12	24	33.48926	2990.748	1345.837	4.25649	Yes
12	25	15.6358	419.1053	167.6421	6.94427	Yes
12	26	51.92262	4621.643	1848.657	6.94427	Yes
12	27	7.88516	106.5869	42.63477	6.94427	Yes
12	28	16.8301	226.6019	75.53397	19	Yes

12	29	47.57256	7040.908	3300.426	3.68232	Yes
13	14	6.96468	32.33781	0	NA	NA
13	15	4.63737	14.33678	0	NA	NA
13	16	2.03957	2.77323	0	NA	NA
13	17	3.74898	9.36988	0	NA	NA
13	18	2.11937	4.49174	1.12294	199.5	No
13	19	0.34354	0.14163	0.04721	19	No
13	20	8.52072	48.40183	0	NA	NA
13	22	8.72846	50.79065	0	NA	NA
13	23	0.40727	0.23695	0.09478	6.94427	No
13	24	3.30495	17.4763	7.64588	4.73741	Yes
13	25	8.47651	86.22142	28.74047	19	Yes
13	26	6.58011	51.95738	17.31913	19	No
13	27	8.25663	81.8063	27.26877	19	Yes
13	28	8.73907	50.9142	0	NA	NA
13	29	10.55007	194.7818	90.43443	3.80557	Yes
14	15	5.7715	16.65513	-Inf	NA	NA
14	16	8.14121	33.13969	-Inf	NA	NA
14	17	15.15346	114.8136	-Inf	NA	NA
14	18	11.94332	95.09519	0	NA	NA
14	19	4.52008	15.32334	3.83083	199.5	No
14	20	13.04884	85.13607	-Inf	NA	NA
14	22	6.34115	20.10508	-Inf	NA	NA
14	23	7.01789	41.04237	15.39089	9.55209	Yes
14	24	19.84001	349.8899	149.9528	5.14325	Yes
14	25	2.05274	3.16031	0.79008	199.5	No
14	26	6.43079	31.01633	7.75408	199.5	No
14	27	19.57271	287.3182	71.82955	199.5	No
14	28	1.73541	1.50582	-Inf	NA	NA
14	29	2.60327	6.32519	2.91932	3.88529	No
15	16	11.5609	66.82717	-Inf	NA	NA
15	17	3.79718	7.2093	-Inf	NA	NA
15	18	12.9493	111.7895	0	NA	NA
15	19	4.75281	16.9419	4.23548	199.5	No
15	20	1.48213	1.09836	-Inf	NA	NA
15	22	18.19161	165.4673	-Inf	NA	NA
15	23	2.55013	5.41928	2.03223	9.55209	No
15	24	12.43318	137.4079	58.8891	5.14325	Yes
15	25	2.22637	3.71754	0.92939	199.5	No
15	26	16.30873	199.4809	49.87022	199.5	No
15	27	4.53873	15.45003	3.86251	199.5	No
15	28	2.65931	3.53597	-Inf	NA	NA
15	29	14.8711	206.4062	95.26442	3.88529	Yes
16	17	11.0121	60.63321	-Inf	NA	NA
16	18	0.5019	0.16794	0	NA	NA
16	19	1.48972	1.66445	0.41611	199.5	No
16	20	18.58781	172.7533	-Inf	NA	NA
16	22	3.48044	6.05674	-Inf	NA	NA
16	23	4.22568	14.88033	5.58012	9.55209	No
16	24	5.56533	27.53143	11.79918	5.14325	Yes
16	25	13.74153	141.6222	35.40554	199.5	No
16	26	1.98598	2.95809	0.73952	199.5	No

16	27	18.37635	253.2677	63.31693	199.5	No
16	28	13.6511	93.17622	-Inf	NA	NA
16	29	7.11812	47.28976	21.82604	3.88529	Yes
17	18	9.6701	62.3406	0	NA	NA
17	19	5.86704	25.81663	6.45416	199.5	No
17	20	2.99309	4.4793	-Inf	NA	NA
17	22	23.70413	280.943	-Inf	NA	NA
17	23	2.05551	3.52095	1.32036	9.55209	No
17	24	4.46095	17.68892	7.58097	5.14325	Yes
17	25	11.50839	99.33228	24.83307	199.5	No
17	26	20.20912	306.3064	76.57659	199.5	No
17	27	1.01768	0.77676	0.19419	199.5	No
17	28	12.34552	76.20598	-Inf	NA	NA
17	29	24.64198	566.7455	261.5748	3.88529	Yes
18	19	2.34887	6.62061	2.20687	19	No
18	20	18.92973	238.8897	0	NA	NA
18	22	6.37841	27.12275	0	NA	NA
18	23	4.31444	26.59205	10.63682	6.94427	Yes
18	24	3.10536	15.42925	6.7503	4.73741	Yes
18	25	17.20621	355.2646	118.4215	19	Yes
18	26	4.26139	21.79133	7.26378	19	No
18	27	16.92184	343.6186	114.5395	19	Yes
18	28	17.28583	199.1999	0	NA	NA
18	29	11.39897	227.3889	105.5734	3.80557	Yes
19	20	9.84061	72.62817	18.15704	199.5	No
19	22	5.99924	26.99313	6.74828	199.5	No
19	23	1.0023	1.88365	0.78486	5.78614	No
19	24	5.49751	65.94024	29.30677	4.45897	Yes
19	25	6.86607	70.71439	26.5179	9.55209	Yes
19	26	4.39108	28.9224	10.8459	9.55209	Yes
19	27	10.86935	177.2141	66.45528	9.55209	Yes
19	28	6.9558	36.28736	9.07184	199.5	No
19	29	7.0948	124.36	58.03468	3.73889	Yes
20	22	29.24148	427.532	-Inf	NA	NA
20	23	5.20763	22.59947	8.4748	9.55209	No
20	24	14.25582	180.6473	77.42029	5.14325	Yes
20	25	6.41729	30.88622	7.72155	199.5	No
20	26	26.43788	524.221	131.0553	199.5	No
20	27	1.67075	2.09355	0.52339	199.5	No
20	28	7.22255	26.08262	-Inf	NA	NA
20	29	25.70133	616.521	284.5482	3.88529	Yes
22	23	11.89875	117.9835	44.2438	9.55209	Yes
22	24	17.83871	282.8617	121.2264	5.14325	Yes
22	25	14.97312	168.1457	42.03642	199.5	No
22	26	0.21276	0.03395	0.00849	199.5	No
22	27	32.92164	812.8758	203.219	199.5	Yes
22	28	14.31801	102.5027	-Inf	NA	NA
22	29	1.68921	2.66322	1.22918	3.88529	No
23	24	4.12194	52.2782	23.76282	4.10282	Yes
23	25	6.72959	84.9138	35.38075	5.78614	Yes
23	26	9.57681	171.966	71.65252	5.78614	Yes
23	27	5.28065	52.28478	21.78533	5.78614	Yes

23	28	7.11924	42.23629	15.83861	9.55209	Yes
23	29	12.61259	586.075	275.8	3.63372	Yes
24	25	21.35507	994.9941	442.2196	4.45897	Yes
24	26	14.20033	439.9625	195.5389	4.45897	Yes
24	27	9.29392	188.4588	83.75948	4.45897	Yes
24	28	21.98674	429.7038	184.1588	5.14325	Yes
24	29	23.67978	2854.637	1355.952	3.52189	Yes
25	26	14.45688	313.502	117.5633	9.55209	Yes
25	27	13.04818	255.3824	95.76838	9.55209	Yes
25	28	0.02456	0.00045	0.00011	199.5	No
25	29	9.27488	212.5283	99.17988	3.73889	Yes
26	27	29.07452	1267.992	475.497	9.55209	Yes
26	28	13.93552	145.6491	36.41227	199.5	No
26	29	2.51869	15.67288	7.31401	3.73889	Yes
27	28	14.10705	149.2566	37.31415	199.5	No
27	29	32.28817	2575.652	1201.971	3.73889	Yes
28	29	8.55849	68.36455	31.55287	3.88529	Yes

[a] Significance at $\alpha = 0.05$.

Numbers are rounded to the first 5 decimal positions.

Supplementary File S7. Distribution of characters involved in plant-bacteria interaction within the 121 genomes of the P. corrugata subgroup.

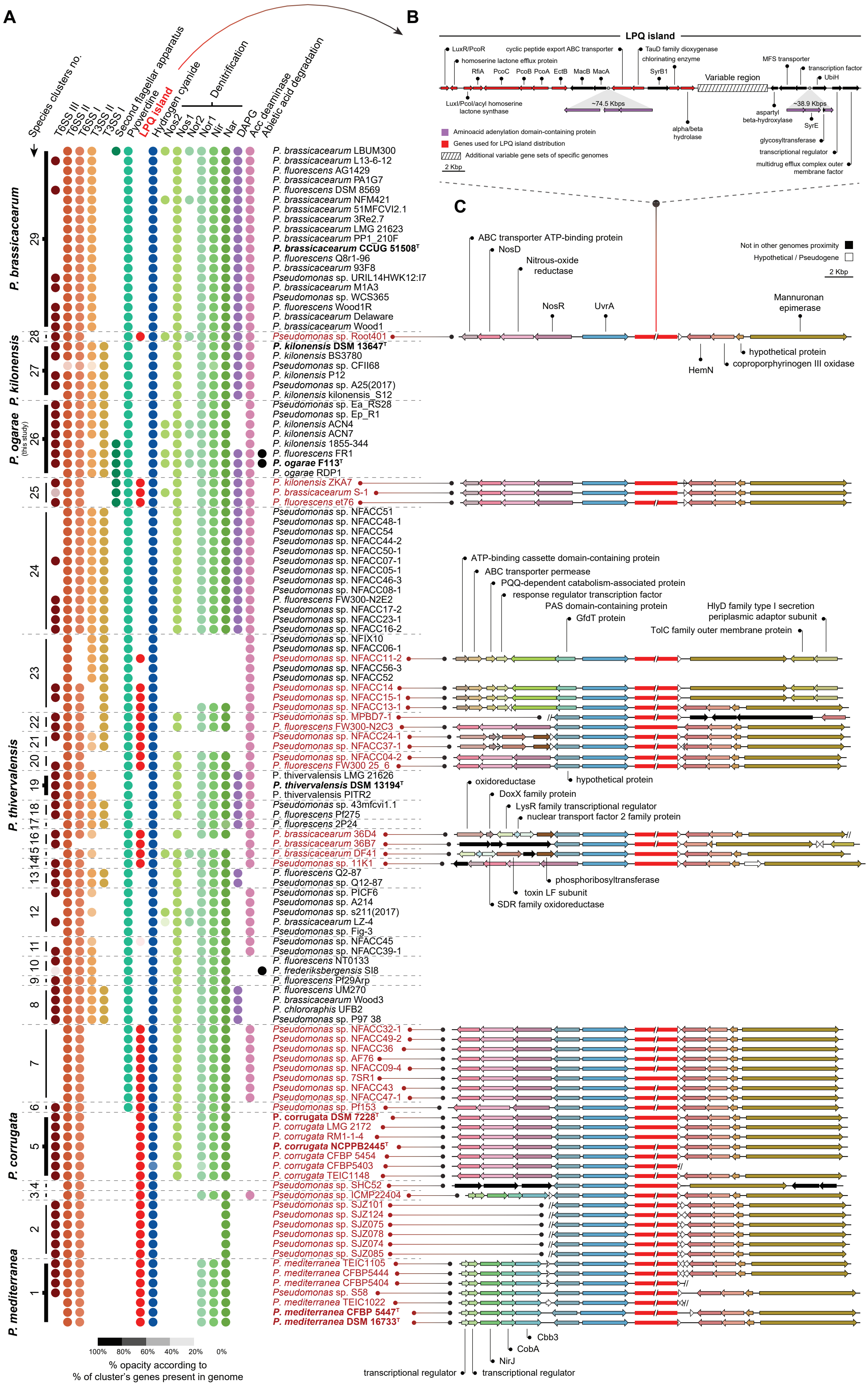

(Fig. S1. caption in next page)

Fig. S1. (A) Distribution of relevant plant-interaction traits among *P. corrugata* subgroup genomes and **(B)** map of the LPQ island and syntenic organization of its flanking regions in each genome. Distribution of characters is based on the identification of orthologous groups of a discrete number of proteins, given that others belong to larger homologous groups not exclusive of the trait analyzed. The proteins used for each trait and are: T6SS III (ImpA3B3C3F2G3H3I3J3K3 + TssE2 + VasD2/TssJ + ClpB4/TssH), T6SS II (ImpA2B2C2D2F2G2H2IJ2KM + VasD/TssJ), T6SS I (ImpABCDFGHJKL + TssJ3), T3SS II (HrcJNQRUV + [illegible] + SlaADEFGHIJLMOPQSTVZ), T3SS I (InvAEF + HilA + PrgHIJK + IagB + OrgAB + SpaLMNOPS + SipBD + YopD + IacP), Second flagellar apparatus (FlgA2B2C2D2E2F2G2H2I2J2K2L2M2N2 + MotA2B2CD + FlbD + FlhCF2 + FliA2D2E2F2G2H2I2J2K2L2M2O2P2Q2S2T2 + O-linked N-acetylglucosamine), Pyoverdine (PvdLAONMSP), LPQ island (PcoABCIR + RhtB + RfiA + pimeloyl-ACP methylester carboxylesterase + SyrP + transport protein + diaminobutyrate-2-oxoglutarate + halogenase), Hydrogen cyanide (HcnABC), Nos2 (NosD2L2R2Y2), Nos1 (NosD1F1L1R1Y1Z1), Nor2 (NorB2C2D2), Nor1 (NorB1C1D1), Nir (NirCDEFGHJLMNQS), Nar (NarGHIJKLUX), DAPG (PhlABCDEFGH), Acc deaminase (AcdS), Abietic acid degradation (DitA1A2A3BCDGHIJKLMOPR). Genomes in red indicate those harboring the LPQ island. Genomes of type strains in bold and T. Species cluster number according to Figure 1.

Supplementary File S9. Phenotypic characteristics of *P. ogarae*, *P. kilonensis*, *P. brassicacearum* and *P. thivervalensis* strains using Biolog GEN II MicroPlate™, API® 20 NE and API® 50 CH tests.

Test	*P. ogarae* F113^T	*P. ogarae* RDP1	*P. kilonensis* DSM 13647^T	*P. brassicacearum* DSM 13227^T	*Pseudomonas* sp. WCS365	*P. brassicacearum* NFM421	*P. fluorescens* Q8r1-96	*P. thivervalensis* DSM 13194^T
**Biolog Gen III MicroPlate (assimilation)**								
Negative Control	-	-	-	-	-	-	-	-
Dextrin	-	-	-	-	-	-	-	-
D-Maltose	-	-	-	-	-	-	-	-
D-Trehalose	+	+	+	+	+	+	+	+
D-Cellobiose	-	-	-	-	-	-	-	-
Gentiobiose	-	-	-	-	-	-	-	-
Sucrose	+	+	+	+	+	+	+	+
D-Turanose	-	-	-	-	-	-	-	-
Stachyose	-	-	-	-	-	-	-	-
D-Raffinose	-	-	-	-	-	-	-	-
α-D-Lactose	-	-	-	-	-	-	-	-
D-melibiose	-	-	-	-	-	-	-	-
β-Methyl-D-Glucoside	-	-	-	-	-	-	-	-
D-Salicin	-	-	-	-	-	-	-	-
N-Acetyl-D-Glucosamine	-	-	-	+	-	+	+	-
N-Acetyl-β-D-Mannosamine	-	-	-	-	-	-	-	-
N-Acetyl-D-Galactosamine	-	-	-	-	-	-	-	-
N-Acetyl Neuraminic Acid	-	-	-	-	-	-	-	-
α-D-Glucose	+	+	+	+	+	+	+	+
D-Mannose	+	+	+	+	+	+	+	+
D-Fructose	-	-	+	+	-	+	+	+
D-Galactose	+	+	+	+	+	+	+	+
3-Methyl Glucose	-	-	-	-	-	-	-	-
D-Fucose	-	-	-	-	-	-	-	-
L-Fucose	-	-	-	-	-	-	-	-
L-Rhamnose	-	-	-	-	-	-	-	-
Inosine	+	+	+	+	-	+	+	-
D-Sorbitol	+	+	+	+	+	+	+	+
D-Mannitol	+	+	+	+	+	+	+	+
D-Arabitol	+	+	+	+	-	+	-	-
myo-Inositol	+	+	+	+	+	+	+	+
Glycerol	+	+	+	+	+	+	+	+
D-Glucose-6-PO4	-	-	-	-	-	-	-	-
D-Fructose-6-PO4	-	-	-	-	-	-	-	-
D-Aspartic acid	+	+	-	+	+	+	+	+
D-Serine	-	-	-	-	-	-	-	-
Gelatin	-	-	-	-	-	-	-	-
Glycyl-L-Proline	-	-	-	-	-	-	-	-
L-Alanine	+	+	+	+	+	+	+	+
L-Arginine	+	+	+	+	+	+	+	-
L-Aspartic acid	+	+	+	+	+	+	+	+

L-Glutamic acid	+	+	+	+	+	+	+	+
L-Histidine	-	+	+	+	+	+	-	-
L-Pyroglutamic acid	+	+	+	+	+	+	+	+
L-Serine	-	-	-	+	-	+	-	-
Pectin	+	+	+	-	-	+	-	-
D-Galacturonic acid	-	+	-	+	+	+	+	-
L-Galacturonic acid Lactone	-	-	-	+	+	+	+	-
D-Gluconic acid	+	+	+	+	+	+	+	+
D-Glucuronic acid	-	+	-	+	+	+	+	-
Glucuronamide	-	-	-	-	-	-	-	-
Mucic acid	+	+	+	+	+	+	+	+
Quinic acid	+	+	+	+	+	+	+	+
D-Saccharic acid	+	+	+	+	+	+	+	+
p-Hydroxy-phenylacetic acid	+	+	+	+	+	+	+	+
Methyl pyruvate	+	+	+	+	+	+	+	+
D-Lactic acid Methyl Esther	-	-	-	-	-	-	-	-
L-Lactic Acid	+	+	+	+	+	+	+	+
Citric acid	+	+	+	+	+	+	+	+
α-Keto-Glutaric acid	+	+	+	+	+	+	+	+
D-Malic acid	-	-	-	-	-	-	-	-
L-Malic acid	+	+	+	+	+	+	+	+
Bromo-Succinic Acid	+	+	+	+	+	+	+	+
Tween 40	+	+	-	+	-	+	+	+
γ-Amino-Butyric acid	+	+	+	+	+	+	+	+
α-Hydroxy-Butyric acid	-	-	-	-	-	-	-	-
β-Hydroxy-D,L-Butyric acid	+	+	+	+	+	+	+	+
α-Keto-Butyric Acid	-	-	-	-	-	-	-	-
Acetoacetic acid	-	-	-	-	-	-	-	-
Propionic acid	-	-	-	-	-	-	-	-
Acetic acid	+	+	+	+	-	-	-	-
Formic acid	-	-	-	-	-	-	-	-
**Biolog Gen III MicroPlate (sensitivity)**								
Positive Control	+	+	+	+	+	+	+	+
pH6	+	+	+	+	+	+	+	+
pH5	-	-	-	-	-	+	+	-
1% NaCl	+	+	+	+	+	+	+	+
4% NaCl	-	-	-	-	-	-	-	-
8% NaCl	-	-	-	-	-	-	-	-
1% Sodium Lactate	-	-	-	+	-	+	+	+
Fusidic acid	-	-	-	-	-	-	-	-
D-Serine	-	-	-	-	-	-	-	-
Troleandomycin	-	-	-	-	-	-	-	+
Rifamycin SV	+	+	+	+	+	+	+	+
Minocycline	-	-	-	-	-	-	-	-
Lincomycin	+	+	+	+	+	+	+	+
Guanidine HCl	-	-	+	-	-	-	-	+
Niaproof 4	+	-	-	-	-	+	+	-
Vancomycin	+	+	+	+	+	+	+	+
Tetrazolium Violet	+	+	+	+	+	+	+	+
Tetrazolium Blue	+	+	+	+	+	+	+	+
Nalidixic Acid	-	-	-	-	-	-	-	-
Lithium Chloride	-	-	-	-	-	-	-	-
Potassium Tellurite	+	+	+	+	-	+	+	+
Aztreonam	-	-	+	-	-	+	-	+
Sodium butyrate	-	-	-	-	-	-	-	-
Sodium Bromate	-	-	-	-	-	-	-	-
**API 20 NE**								
Reduction of nitrates	+	+	w	-	+	w	w	-
Indole production	-	-	-	-	-	-	-	-
Fermentation (Glucose)	-	-	-	-	-	-	-	-
Arginine dihydrolase	-	-	-	+	+	+	+	+

Urease	-	-	-	-	-	-	-	-
Hydrolysis (β-glucosidase)	+	+	+	+	+	+	+	+
Hydrolysis (protease)	+	+	+	+	-	+	+	+
b-galactosidase	-	-	-	-	-	-	-	-
Glucose assimilation	+	+	+	+	+	+	+	+
Arabinose assimilation	+	+	+	+	+	+	+	+
Mannose assimilation	+	+	+	+	+	+	+	+
Mannitol assimilation	+	+	+	+	+	+	+	+
N-acetyl-glucosamine assimilation	-	-	-	+	+	+	+	+
Maltose assimilation	-	-	-	-	-	-	-	-
Potassium gluconate assimilation	+	+	+	+	+	+	+	+
Capric acid assimilation	+	+	+	+	+	+	+	+
Adipic acid assimilation	-	-	-	-	-	-	-	-
Malate assimilation	+	+	+	+	+	+	+	+
Trisodium citrate assimilation	+	+	+	+	+	+	+	+
Phenylacetic acid assimilation	-	-	-	-	-	-	-	-
**API 50 CH**								
Control	-	-	-	-	-	-	-	-
Glycerol	+	+	w	+	+	+	+	+
Erythrol	-	-	-	-	-	-	-	-
D-arabinose	w	w	-	-	-	-	-	-
L-arabinose	+	+	+	+	+	+	+	+
D-ribose	+	w	w	w	+	+	w	w
D-xylose	+	+	+	+	+	+	+	+
L-xylose	w	-	-	-	-	-	-	-
Adonitol	-	-	-	-	-	-	-	-
Methyl-beta-D-xylopyranoside	-	-	-	-	-	-	-	-
D-galactose	+	+	+	+	+	+	+	+
D-glucose	+	+	+	+	+	+	+	+
D-fructose	+	+	+	+	+	+	+	+
D-mannose	+	+	+	+	+	+	+	+
L-sorbose	-	-	-	-	-	-	-	-
L-rhamnose	-	-	-	-	-	-	-	-
Dulcitol	-	-	-	-	-	-	-	-
Inositol	+	+	w	w	+	+	w	+
D-mannitol	+	+	w	+	+	+	+	+
D-sorbitol	+	+	w	+	+	+	+	+
Methyl-alpha-D-mannopyranoside	-	-	-	-	-	-	-	-
Methyl-alpha-D-glucopyranoside	-	-	-	-	-	-	-	-
N-acetylglucosamine	-	-	-	-	-	-	-	-
Amygdalin	-	-	-	-	-	-	-	-
Arbutin	-	-	-	-	-	-	-	-
Esculin ferric citrate	+	+	+	+	+	+	+	+
Salicin	-	-	-	-	-	-	-	-
D-cellobiose	-	-	-	-	-	-	-	-
D-maltose	-	-	-	-	-	-	-	-
D-lactose (bovine origin)	-	-	-	-	-	-	-	-
D-melibiose	+	+	w	-	+	+	w	+
D-saccharose (sucrose)	+	+	w	-	+	+	+	+
D-trehalose	+	+	+	w	+	+	+	+
Inulin	-	-	-	-	-	-	-	-
D-melezitose	-	-	-	-	-	-	-	-
D-raffinose	-	-	-	-	-	-	-	-
Amidon (starch)	-	-	-	-	-	-	-	-
Glycogen	-	-	-	-	-	-	-	-
Xylitol	-	-	-	-	-	-	-	-
Gentiobiose	w	w	-	-	-	-	-	w
D-turanose	-	-	-	-	-	-	-	-
D-lyxose	+	-	-	-	+	-	-	-
D-tagatose	-	-	-	-	-	-	-	-

D-fucose	+	+	+	+	+	+	+	+
L-fucose	-	-	-	-	-	-	-	-
D-arabitol	+	+	-	w	+	w	w	+
L-arabitol	-	-	-	-	-	-	-	-
Potassium gluconate	-	-	-	-	-	-	-	-
Potassium 2-ketogluconate	-	-	-	-	-	-	-	-
Potassium 5-ketogluconate	-	-	-	-	-	-	-	-

For assimilation and oxidation of carbon compounds: positive (+), negative (-), weakly positive (w). For sensitivity: unsensitive (+, can grow in its presence), sensitive (-, cannot grow in its presence).
